# Impact of cooking with liquefied petroleum gas compared with traditional cooking practices on perinatal and early neonatal mortality: the Poriborton cluster randomised controlled trial

**DOI:** 10.1136/bmjgh-2025-020391

**Published:** 2026-02-16

**Authors:** Camille Raynes-Greenow, Sk Masum Billah, Jonathan Thornburg, Sajia Islam, S M Rokonuzzaman, Neeloy Ashraful Alam, Michelle McCombs, Kingsley Agho, Shams El Arifeen, Michael J Dibley

**Affiliations:** 1 Sydney School of Public Health, The University of Sydney, Camperdown, New South Wales, Australia; 2 RTI International, Research Triangle Park, North Carolina, USA; 3 Maternal and Child Health Division, International Centre for Diarrhoeal Disease Research, Bangladesh (ICDDR,B), Dhaka, Bangladesh; 4 School of Science and Health, Western Sydney University—Campbelltown Campus, Campbelltown, New South Wales, Australia

**Keywords:** Clinical trial, Public Health, Epidemiology

## Abstract

**Objective:**

To determine whether liquefied petroleum gas (LPG) can reduce perinatal mortality in a setting with high reliance on biomass fuels for cooking.

**Design:**

Community-based two-arm parallel cluster randomised controlled trial, in Sherpur, Bangladesh.

**Participants:**

4944 pregnant women were recruited, with 2472 in each group. Eligible women were pregnant between 40 and 120 days, aged 15–49 years, permanent residents and planning to give birth in their cluster of residence.

**Intervention:**

LPG stove and fuel to birth. Controls continued with usual cooking practices.

**Main outcome:**

Primary outcome at the individual level: perinatal mortality. Secondary outcomes: early neonatal mortality, neonatal mortality, preterm birth and personal exposure to particulate matter 2.5 (PM2.5).

**Results:**

The final birth outcomes included 4592 participants. The perinatal mortality rate (PMR) was 50 per 1000 births in the intervention group compared with 61 per 1000 births in the control group (relative risk (RR) 0.83; 95% CI 0.65 to 1.06). PM2.5 concentrations were 47.2 µg/m³ in the intervention versus 60.3 µg/m³ in the control; mean difference −0.133 (95% CI −0.194 to −0.072). In late pregnancy, it was 62·7 µg/m^3^ in the intervention versus 88·7 µg/m^3^ in the control, mean difference −0.149 (−0.198 to –0.101). Early neonatal mortality was 22% in the intervention compared with 30% in the control (RR 0.73; 95% CI 0.50 to 1.05). Preterm birth rates were similar. In post hoc subgroup of small versus large households (HH), the PMR was lower in the smaller HH in the intervention group (67, rate 54 per 1000 births) than in the control group (102, 71 per 1000 births, adjusted RR 0.75; 95% CI 0.56 to 1.00; p=0.047).

**Conclusions:**

Reductions in perinatal mortality favoured the intervention but were statistically non-significant. These findings demonstrate a reduction in mortality in smaller HH when cooking needs are adequately covered by clean fuel.

**Trial registration number:**

ACTRN12618001214224; Australian and New Zealand Clinical Trials Registry.

**WHAT IS ALREADY KNOWN ON THIS TOPIC**

- Evidence from three systematic reviews with meta-analyses reported a harmful effect estimate of household air pollution on perinatal outcomes, specifically stillbirth and low birth weight. The included studies were all observational with limitations and high risk of bias. More recently, evidence from a multisite randomised controlled trial (RCT) reported no effect on stillbirth or neonatal mortality.

**WHAT THIS STUDY ADDS**

- This single site cluster RCT is the first to assess perinatal mortality as the primary outcome. The evidence indicates a reduction in perinatal mortality associated with cooking with liquefied petroleum gas (LPG) compared with usual cooking practices but was not statistically significant. In subgroup analyses of smaller-sized households, the perinatal mortality rate was significantly lower in the intervention group compared with the control.

**HOW THIS STUDY MIGHT AFFECT RESEARCH, PRACTICE OR POLICY**

- These findings demonstrate that cooking with LPG during pregnancy can reduce personal exposure to particulate matter 2.5 and reduce the risk of perinatal mortality in households with fuel security and who used clean fuels exclusively. Infrastructure to support community adoption of clean cooking is needed to support fuel security in settings that rely on biomass fuel, and this will contribute to infant survival.

## Background

 Despite global reductions in perinatal mortality, global stillbirth and early neonatal mortality burden remain high at an estimated 3.6 million perinatal deaths every year.^1 2^ Most of these deaths occur in low and middle-income countries, especially in sub-Saharan Africa and South Asia.^1^,^3^ Similarly, countries in these two regions continue to have the highest burden of exposure to household air pollution (HAP).^4^ HAP is primarily due to cooking and heating with polluting fuel and is recognised as one of the leading risk factors for the burden of disease globally.^5 6^ Evidence of the risk of exposure from HAP on the developing fetus is primarily based on the impact of similar exposures such as environmental tobacco smoke and ambient air pollution. In 2006, the WHO identified the need for evaluation of the effects of HAP on pregnancy outcomes.^7^

Epidemiological evidence for the effect of HAP on adverse pregnancy outcomes has been increasing but has been based mostly on retrospective observational studies.^8 9^ There have been two systematic reviews with meta-analyses to produce summary effects of HAP on stillbirth and other pregnancy outcomes.^10 11^ The estimated increase in the odds of stillbirth in these two reviews was reported as 51% and 29%.^10 11^ However, this evidence has been undermined by the methodological weakness of the included studies, which have been previously described.^10^,^12^ Intervention trials on perinatal mortality using liquefied petroleum gas (LPG), biofuels or improved stove designs had not been conducted when we commenced this trial. As a cleaner, efficient fuel, LPG is portable, safe, lower cost and does not need major infrastructure for the user and is ideal for low and middle-income settings.^13 14^ Recently, a multicountry trial of LPG or biomass for cooking on the effect on birth weight was published with no effect and a null finding, it was not powered for perinatal mortality.^15 16^

Our aim was to assess the impact of reducing HAP exposure from early pregnancy to birth by using LPG for cooking, compared with usual cooking methods, on perinatal mortality, stillbirth and both early and late neonatal mortality. We hypothesised that the use of LPG for cooking would reduce perinatal mortality (stillbirth and early neonatal mortality) by 35% compared with usual cooking practices.

## Methods

We conducted a community-based two-arm parallel cluster randomised controlled trial, called ‘Poriborton’ in which we supplied LPG stoves and cylinders for cooking (intervention) to pregnant women and compared this to usual cooking practices (control). Poriborton is a Bangla word that means ‘change’. The trial was conducted in Sherpur district, of the Mymensingh Division of Northern Bangladesh. Poriborton was registered with the Australian and New Zealand Clinical Trials Registry (ACTRN12618001214224), and the protocol has been previously published, which includes more detail about the study procedures.^17^

### Participants

The study population consisted of married women who were residents of Sherpur District. Pregnancy out of marriage is socially unacceptable, so all pregnant women are married. There were two levels of eligibility, the first for the clusters that were predefined geographical areas, and the second for resident women of reproductive age. Clusters were eligible if they were predominantly rural and excluded if (1) they had already participated in a stove scheme or (2) were within or sharing boundaries of areas with a relatively high existing use of LPG cooking or (3) were prone to extended periods of flooding.^17^ Eligible women were (1) aged between 15 and 49 years, (2) were pregnant (urine test confirmed) with a gestational age between 40 and 120 days determined by self-report of the date of last menstrual period (LMP), (3) permanent residents of the study area and (4) planning to give birth in their cluster of residence. The exclusion criteria were: (1) women whose LMP date could not be determined and (2) those who had a pregnancy loss before receiving the intervention.

Recruitment was rolling and occurred through door-to-door surveillance of the LMP of all eligible women in the households in the study areas. Community leaders and relevant government officials provided gatekeeper consent.

### Randomisation and masking

We used census data and the smallest administrative level (unions) in the Mymensingh Division to estimate cluster sizes. We estimated ~25 000 people per union, and then divided these into areas of ~445 households, which formed the basis of the clusters. We then conducted household enumeration surveys and listed all the residents to determine the exact population size. The 206 clusters were then block randomised, in a 1:1 ratio using Stata SE (StataCorp V.2023. Stata V.18. Statistical software. StataCorp LLC). Randomisation was performed by research staff who were not involved in the study. The intervention was distributed at the individual level, whereas randomisation occurred at the cluster level (online supplemental figure 1).

The nature of the intervention prevented blinding, or masking, hence, we ensured that our field staff were blinded to the study hypothesis and were separated into teams with different roles. One team conducted surveillance and screened for pregnancy, a second team enrolled participants and collected all follow-up data but were not involved in any intervention implementation, and a third team delivered the intervention including behaviour change communication. Data analysis was conducted by an independent statistician who was blinded to treatment allocation.

### Procedures

We conducted household surveillance of the married women’s LMP to screen for eligibility. When an eligible woman was identified, her pregnancy was confirmed using a urine test, and participation was discussed. The surveillance team then recorded the eligible participants in the web-linked electronic data collection system, which alerted the data collection team to obtain consent and enrol eligible women. Participants in the intervention arm received the intervention within 7 days of enrolment. Data were collected at six time points, the first three in each pregnancy trimester, another following the birth, a fifth up to 10 days postpartum and the final at 45 days postpartum.^17^ Data were self-reported and verified using medical records and death certificates if possible.

The intervention comprised of provision of a double-burner LPG stove and a 15 kg LPG cylinder that met the Bangladeshi National Safety Standards. The cylinder was replaced as needed to ensure continuous fuel supply throughout pregnancy until the birth of the infant. All provided at no cost to participants. Participants and households received behaviour change communication to support exclusive LPG cooking, which was presented monthly via household visits. The behaviour change communication strategy included a three-part video story, a pictorial wall calendar (online supplemental file 1 calendar), and a pictorial leaflet (online supplemental file 2 leaflet). All were embedded with behaviour change messages developed from our previous work,^17^ except for the pictorial leaflet, which included only safety messages. The monthly household visits occurred throughout pregnancy to support LPG cooking and provide troubleshooting. Control participants continued with usual cooking practices and did not receive an intervention; however, they received a small gift of ~US$2 value when they notified the field staff of the birth.

The primary outcome was the perinatal mortality rate, calculated as the sum of the number of stillbirths (fetal death in pregnancy, before or during birth ≥28 weeks gestation) and the number of early neonatal deaths (death of a live-born infant <7 days after birth, regardless of gestational age or birth weight) per 1000 births at the individual level. Notification of birth outcomes was prompted by mobile phone reminders to the participants or captured via scheduled household visits. Almost all pregnancy outcomes were verified by a household visit from our data collection team, and where possible, deaths were verified by health facility records. Neonatal death was defined as death of a liveborn infant from birth to ≤28 completed days after birth. Gestational age was determined using the LMP. We conducted two rounds of monthly LMP surveillance in our study population before we commenced recruitment to ensure that we were recruiting incident pregnancies and to improve the estimation of gestational age, as ultrasound for gestational age dating is not routinely performed in this setting. If an ultrasound had been used to determine gestational age, this was used for analysis. Preterm birth was defined as a live birth before 37 completed weeks gestation, or in the absence of gestation age, birth weight <2500 g if available. Household size was defined as small (≤4 household members) or large (≥5 household members). Maternal body mass index (BMI) at baseline was calculated using standard formula from maternal height and weight, collected at baseline. Household wealth was estimated using standard methods from a structured checklist of assets, which consisted of items on agricultural and homestead land, building materials of dwellings, livestock, water sources, sanitation facilities, electricity connection, furniture and motorised and non-motorised vehicles.^18^ We conducted monthly visits of the intervention households for monitoring stove use, using self-report and kitchen observation. In monthly visits, we asked if the household used stoves other than LPG since the last visit/month and our field staff conducted kitchen observations, including presence of other stoves and evidence of recent biomass stove use. For both intervention and control groups, we collected stove use by 24-hour recall at three time points during the intervention period (enrolment visit, second visit ~5–6 pregnancy months and third visit ~8–9 pregnancy months). These approaches were based on our previous research.^19^

The reduction in HAP exposure resulting from the LPG intervention was measured in a randomly selected subset of the same 150 women per arm during each trimester.^20^,^22^ The MicroPEM (RTI International, Research Triangle Park, North Carolina, USA) worn in the chest pocket of a specially developed sash, measured exposure to respirable particulate matter (with an aerodynamic diameter of ≤2.5 μg/m^3^ (PM_2.5_)) for 24 hours.^20^ Continuous (10 s) and average PM_2.5_ exposure were measured. Mean and median PM2.5 exposures were determined via gravimetric analysis of the MicroPEM’s internal sample filter.^23^

### Statistical analysis

We estimated that we would need to recruit 4944 pregnant women based on a 35% reduction in the perinatal mortality rate in the intervention arm compared with the expected 50 perinatal deaths per 1000 births in the control arm. This was based on a two-sided alpha set at <0.05, powered at 80%, cluster size of 20, with an intracluster correlation of 0.000275 due to the cluster trial design and inflated for losses (detailed description provided elsewhere).^17 24^

The primary comparison was the perinatal mortality rate in the intervention and the control arm. Analysis of secondary outcomes included the effect of LPG cooking on stillbirth, early neonatal death, neonatal death, preterm birth and personal exposure to PM_2.5_.

We analysed the effect of the intervention (LPG cooking) compared with usual cooking practice on the primary and secondary outcomes based on the intention-to-treat principle and according to the analysis plan in the protocol registration.^17^ We excluded pregnancies that ended before 28 completed weeks' gestation, any maternal deaths before the birth and women who migrated out of the study area for whom we could not collect information on pregnancy outcomes after making several attempts. We analysed exposure to PM_2.5_ among a subset of participants for whom exposure data were collected and had a valid measurement.

We calculated the mean and SD of the participants’ characteristics for continuous variables and proportions for binary or categorical variables. Household wealth was calculated using principal component analysis of the asset items to generate a composite asset score for each household, categorised into five relative wealth quintiles, from richest to poorest.^18^

For PM_2.5_ data, we calculated the mean and SD, and median and IQR to account for the log normal distribution. We reported perinatal mortality and stillbirth rates per 1000 births and neonatal mortality per 1000 live births. We employed multivariable logistic binomial regression models with log-link function and generalised estimating equations with exchangeable correlation matrix to estimate the effects of the intervention on primary and secondary outcomes. We included clusters as random effect in the models to adjust for the cluster randomised design. All results were presented using relative risks (RR) or adjusted RRs (aRR) and their 95% CIs.

We conducted analyses of the intervention effect on the outcomes, adjusting for potential confounding by participant characteristics that differed between intervention and control groups at baseline. We tested potential interactions of selected participant characteristics and birth characteristics on mortality outcomes and analysed the intervention effect on perinatal and early neonatal mortality in different participant and birth characteristic subgroups. Statistical analyses were conducted in Stata V.18.0 and plots were prepared to present subgroup analyses in R, V.4.3.1 (The R Foundation for Statistical Computing).

This study was funded by a grant from the National Health and Medical Research Council (GNT1127074), which did not have any role in study design, data collection, data analysis, data interpretation or report writing. The LPG gas contractor also did not play any role in study design, data collection, data interpretation or report writing.

Our trial was conducted during the acute phase of the SARS-CoV-2 pandemic. The Government of Bangladesh instigated a movement restriction intervention from 26 March 2020. At that time, we had finished our fifth round of married women’s LMP surveillance and had recruited 929 (38.58%) participants; however, we then stopped surveillance and recruitment. We advocated that LPG fuel delivery was an essential service; subsequently, fuel delivery was exempted from the movement restrictions, and we were able to continue LPG supply to the intervention arm, with no impact on supply. Field activities resumed on 9 August 2020, but recruitment was slow due to the eligibility criteria for incident pregnancies. We made several minor changes to the protocol and data collection procedures during the SARS-CoV-2 pandemic lockdowns. We changed the monthly household visits and LMP surveillance to mobile phone calls. Data related to cookstove observation in the intervention households could not be collected during lockdowns. We developed and implemented infection prevention and control protocols for each cadre of staff (ie, office staff, surveillance staff, evaluation staff and intervention staff) and a comprehensive personal protective equipment guideline, we monitored COVID cases in our area and adhered to all government mandates.

### Participant and public involvement

Community members were involved in understanding the cultural and practical issues around cooking and adopting new cooking fuels, they also assisted in developing the behaviour change communication and providing support to conduct the study. Field staff were recruited from the community and provided feedback on the delivery and behaviour change messages which were used to tailor the intervention.^19 25^

## Results

**Figure 1:**
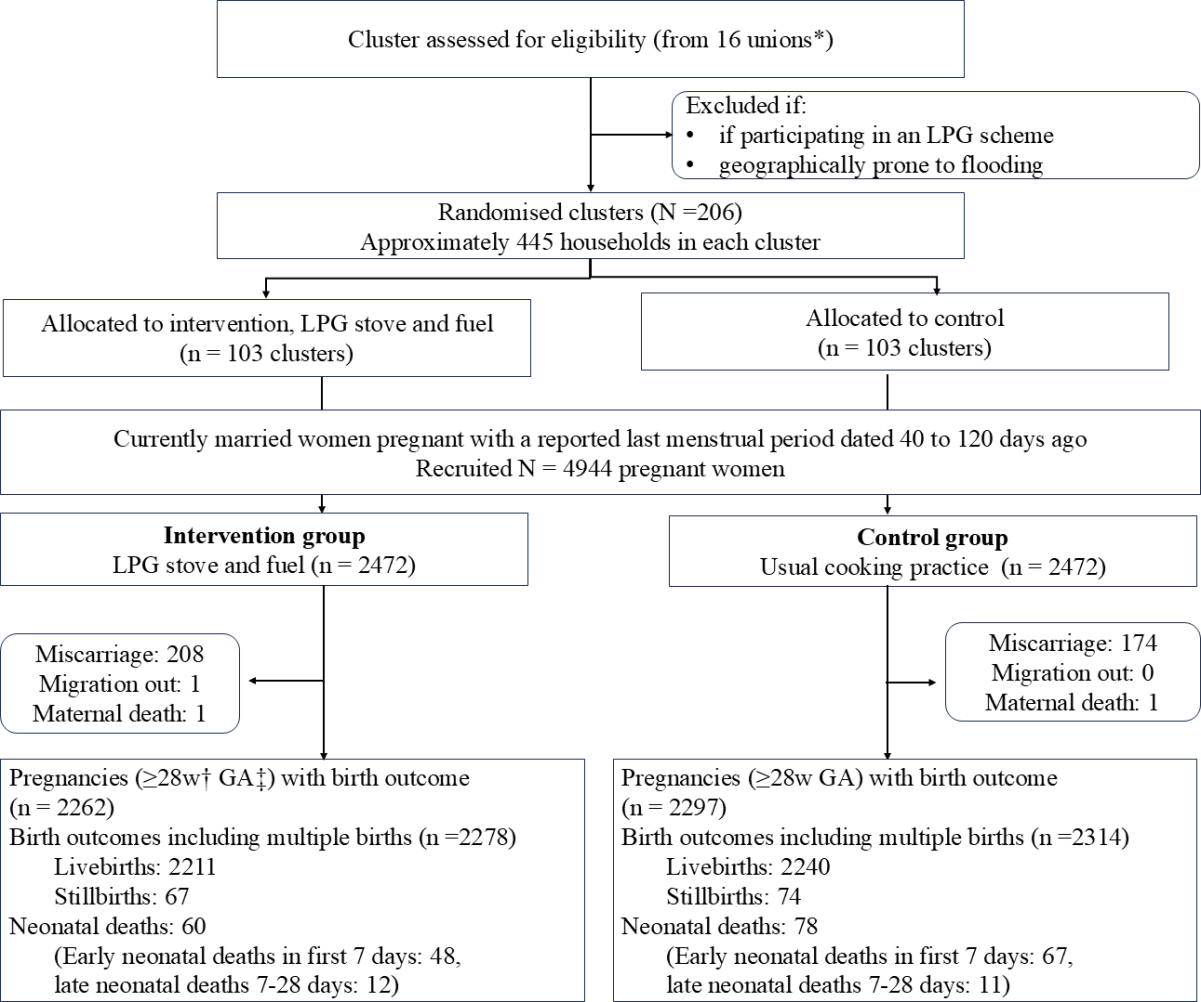
Poriborton trial, flow diagram. *Unions are the lowest administrative unit consisting of ∼ 30 000 people. †w, weeks; ‡GA, gestational age. LPG, liquefied petroleum gas.

We recruited 4944 pregnant women, from 15 rounds of pregnancy surveillance in 206 clusters, between 24 September 2019 and 7 March 2020. Based on their cluster of residence, they were randomised to receive an LPG stove and replacement gas cylinders (n=2472) or continue with their usual cooking practices (n=2472), until the birth of their infant. Follow-up was completed, with only one participant for whom we could not determine the pregnancy outcome due to migration out of the study area. There was one maternal death in each group, foetal loss before 28 weeks was excluded as per the protocol and was higher in the intervention group (n=208) compared with the control group (n=174). The final sample for analysis was 4559 women (2262 in the intervention group and 2297 in the control group) who had 4592 infants (figure 1).

The baseline characteristics of the participating women were balanced between the two groups except maternal age and household size (table 1). For maternal age, although there was no overall difference in the mean age of women between the two groups, there was a small difference in the age distribution, with more younger women (<20 years) in the intervention arm 543/2472 (22%) compared with the control 465/2472 (18.8%). There was also a small difference in mean household size between the two groups, with the intervention group having slightly more mean persons per household (4.6 (SD 1.6)) compared with 4.3 (SD 1.7)) in the control group. There was no difference in education levels, household work as the main occupation, participants’ BMI, mean haemoglobin and mean gestational age in weeks at enrolment and time between identification of a pregnancy and enrolment into the study between the intervention and control group. Median time between enrolment to receiving the intervention was 4 days (IQR 2, 6 days). Parity was evenly distributed in both groups. At baseline, most women (98%) were using traditional clay stoves and biomass fuel as their primary fuel intervention 2435/2472 (98.5%) and 2432/2472 (98.2%) in the control arm. Approximately 60% of households included smokers in both groups.

**Table 1:** Baseline characteristics of participants in the Poriborton trial

Baseline characteristics	Intervention N=2472	Control N=2472
Cluster—n	103	103
Cluster mean (SD)	24.2 (1.93)	24.4 (3.07)
Woman’s age (years)*—n (%)		
<20	543 (22.0)	465 (18.8)
20–24	757 (30.6)	787 (31.8)
25–29	657 (26.6)	739 (29.9)
>30	515 (20.8)	481 (19.5)
Women’s education—n (%)		
No schooling	273 (11.0)	256 (10.4)
Primary (1–5 years)	765 (30.9)	819 (33.1)
Secondary (6–10 years)	1087 (44.0)	1087 (44.0)
Higher secondary or more (≥11 years)	347 (14.0)	310 (12.5)
Women’s main occupation—n (%)		
Household work	2297 (92.9)	2304 (93.2)
Paid employment	175 (7.1)	168 (6.8)
Women’s body mass index		
Mean±SD in kg/m^2^	22.3±4.82	22.3±4.82
Haemoglobin level		
Mean±SD in g/dL	11.2±1.28	11.1±1.26
Missing	59	39
Gestational age at enrolment		
Mean±SD in weeks	11.0±2.74	10.8±2.58
Parity—n (%)		
0	825 (33.4)	739 (29.9)
1	772 (31.2)	800 (32.4)
2 or more	875 (35.4)	933 (37.7)
Previous history of stillbirth—n (%)		
Yes	129 (5.2)	141 (5.7)
No	2343 (94.8)	2331 (94.3)
Previous history of child death—n (%)		
Yes	178 (7.2)	180 (7.3)
No	2294 (92.8)	2292 (92.7)
Household size* (persons)		
Mean±SD	4.6±1.64	4.3±1.65
≤4	1344 (54.4)	1544 (62.5)
≥5	1128 (45.6)	928 (37.5)
Household wealth quintiles—n (%)		
Lowest (poorest)	482 (19.5)	507 (20.5)
Second	507 (20.5)	482 (19.5)
Middle	489 (19.8)	500 (20.2)
Fourth	513 (20.8)	477 (19.3)
Highest	481 (19.5)	506 (20.5)
Primary stove—n (%)		
Traditional (clay made)	2397 (97.0)	2369 (95.8)
Improved cookstove (with or without chimney)	2 (0.1)	5 (0.2)
Biogas stove	0 (0.0)	2 (0.1)
Electric stove	4 (0.2)	5 (0.2)
LPG stove	69 (2.8)	91 (3.7)

* P<0.05.

LPG, liquefied petroleum gas.

The primary outcome, perinatal mortality rate was 50 per 1000 births in the intervention arm compared with 61 per 1000 births in the control arm (RR 0.82; 95% CI 0.64 to 1.05, p=0.120). This included 67 stillbirths and 48 early neonatal deaths (115/2278) compared with 74 stillbirths and 68 early neonatal deaths in the control arm (142/2314) (table 2). The analysis adjusted for baseline differences in maternal age and household size did not alter the results.

**Table 2:** Rate, relative and adjusted risk of LPG cooking compared with usual cooking practices on perinatal outcomes in the Poriborton trial

Outcome	Intervention n/N	Control n/N	Relative risk (95% CI)	P value	Adjusted relative risk* (95% CI)	P value
	(rate per 1000 as specified)					
Primary outcome						
Perinatal mortality†	115/2278 (50)	141/2314 (61)	0.83 (0.65 to 1.06)	0.133	0.83 (0.65 to 1.06)	0.133
Secondary outcomes						
Stillbirth†	67/2278 (29)	74/2314 (32)	0.92 (0.67 to 1.27)	0.610	0.92 (0.67 to 1.27)	0.621
Early neonatal mortality‡	48/2211 (22)	67/2240 (30)	0.73 (0.50 to 1.05)	0.085	0.73 (0.51 to 1.05)	0.088
Neonatal mortality‡	60/2211 (27)	78/2240 (35)	0.78 (0.56 to 1.09)	0.140	0.77 (0.55 to 1.07)	0.121
Preterm live birth§ (%)	481/2211 (21.8)	484/2240 (21.6)	1.01 (0.90 to 1.12)	0.900	1.01 (0.90 to 1.12)	0.915
Any preterm birth§ (still and live birth)—(%)	508/2278 (22.3)	509/2314 (22.0)	1.01 (0.91 to 1.13)	0.800	1.01 (0.91 to 1.13)	0.810

* Adjusted for maternal age at baseline and household size.

† Denominator for perinatal mortality and stillbirth and any preterm birth rates include all births ≥28 completed weeks of gestation.

‡ Denominator for preterm birth, early neonatal mortality and neonatal mortality rates include all live births.

§ Preterm live birth: born alive before 37 completed weeks of gestation. Any preterm birth: stillbirths between ≥28 and <37 completed weeks and live births before 37 completed weeks of gestation.

LPG, liquefied petroleum gas.

### Secondary outcomes

We found reductions in all mortality outcomes favouring the intervention although the CIs included a null value (table 2). For stillbirth, the RR was 0.92; 95% CI (0.67 to 1.27); p=0.610. There were fewer early neonatal deaths in the intervention group 48/2211 (22 %), compared with 68/2240 (30%) in the control, RR 0.72; 95% CI (0.50 to 1.03); p=0.120. Neonatal mortality was lower in the intervention group, 60/2211 (27%) compared with 79/2240 (35%) in the control (RR 0.77; 95% CI (0.55 to 1.07); p=0.120). Adjusting for baseline differences in maternal age and household size did not alter the estimates.

Birth characteristics were similar between the two groups, with 1291/2278 (57.7%) births occurring in a home or non-health facility in the intervention group compared with 1360/2314 (58.8%) to the control group (online supplemental table S1). Mode of birth was similar between the two groups. Most births were vaginal, in both the intervention (1623/2278, 71.2%) and control group (1710/2314, 73.9%). Multiple births accounted for a very small proportion, intervention 32/2278 (1.4%) and control 34/2314 (1.5%) and were included in the analysis as excluding multiple births did not change the results. Preterm live birth was not different between the two groups, 481/2211 (21.8%) in the intervention group, compared with 484/2240 (21.6 %) in the control, RR1.01 (0.90 to 1·12). Adjusting for baseline differences in maternal age and household size did not alter the results.

At baseline, stove use data collected from both groups suggests that mixed fuel use (defined as ‘any/some’ use of any clean fuel and any biomass fuel in the previous 24 hours) was 12.6% in the intervention group and 16.8% in the control group (online supplemental table S2). In the second follow-up visit (before the birth), 5.8% control group were exclusively cooking with cleaner fuel compared with 1.6% at baseline, indicating almost 4% adoption of exclusive use of clean fuel in the control group.

The monthly cooking monitoring data in the intervention group suggest that adherence to LPG cooking was high, although there are some differences between self-report and kitchen observation. Nearly all intervention participants at each time point self-reported that they exclusively used LPG data (data not shown); however, kitchen observation data suggest biomass stoves were present in 14.4% to 23.8% of intervention households throughout the intervention period. Availability of traditional stoves in larger households was double (availability 30.8%–37.8%), compared with (13.6%–18.3%) in smaller households.

### Personal PM_2.5_ exposure

At the first time point, PM_2.5_ exposure was successfully completed in 309 women (98%), 238 (77%) at second follow-up and 265 (85%) at third follow-up. Loss-to-follow was due to device failure, early fetal loss, participant refusal and invalid MicroPEM samples.

The exposure data demonstrated the efficacy of the intervention in reducing personal exposure to PM_2.5_ (online supplemental table S3). The first measurement did not achieve the WHO short-term (24 hours) Interim Target 1 (IT-1) and there was no difference in the 24-hour median PM_2.5_ concentrations between the two groups, 101.6 μg/m^3^ (IQR 57.4) in the intervention compared with 83.5 µg/m^3^ (IQR 89.9) in the control; mean difference −0.0012 (−0.0026 to 0.0002); p=0.099.· At the second time point, the intervention group had a median PM_2.5_ concentration of 47.2 µg/m^3^ (IQR 20.8) and achieved the short-term WHO IT-2, compared with 60.3 µg/m^3^ (IQR 54.0) in the control group; mean difference −0.133 (−0.194 to 0.072); p≤0.001. At the final time point, this difference continued but at the short-term WHO IT-1 in the intervention group 62.7 µg/m^3^ (IQR 34.6) compared with 88.7 µg/m^3^ in the control group (IQR 53.7); mean difference −0.149 (−0.198 to 0.101); p≤0.001. These differences represent a 22% and a 29% difference in PM_2.5_ exposure at two time points between the two groups (online supplemental figure 2).

### Subgroup analyses

**Figure 2:**
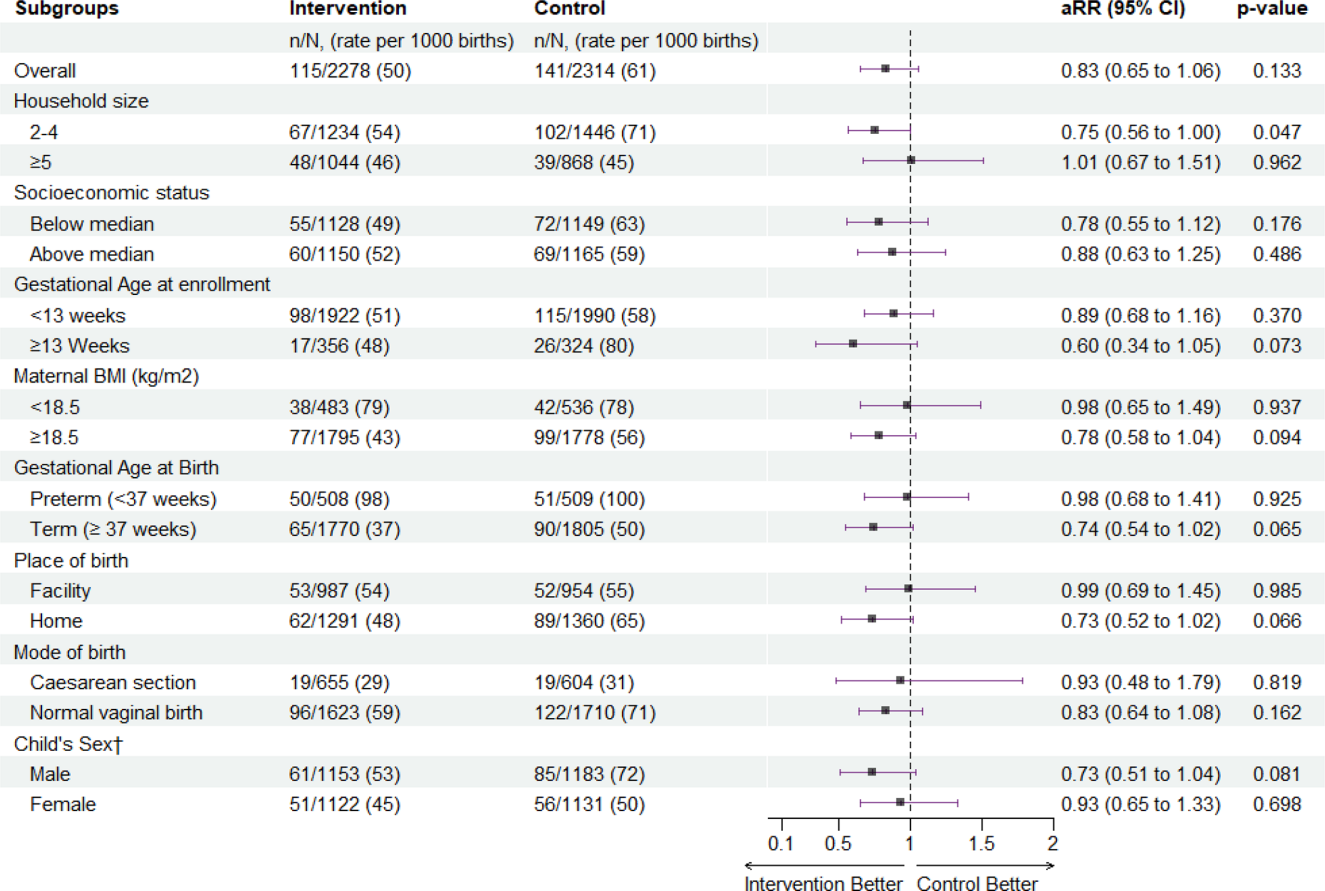
Effect of cooking with LPG on perinatal mortality by subgroups, Poriborton trial. aRR; adjusted relative risk, adjusted for household size and maternal age, †Infant sex missing for three stillbirths. BMI, body mass index; LPG, liquefied petroleum gas.

**Figure 3:**
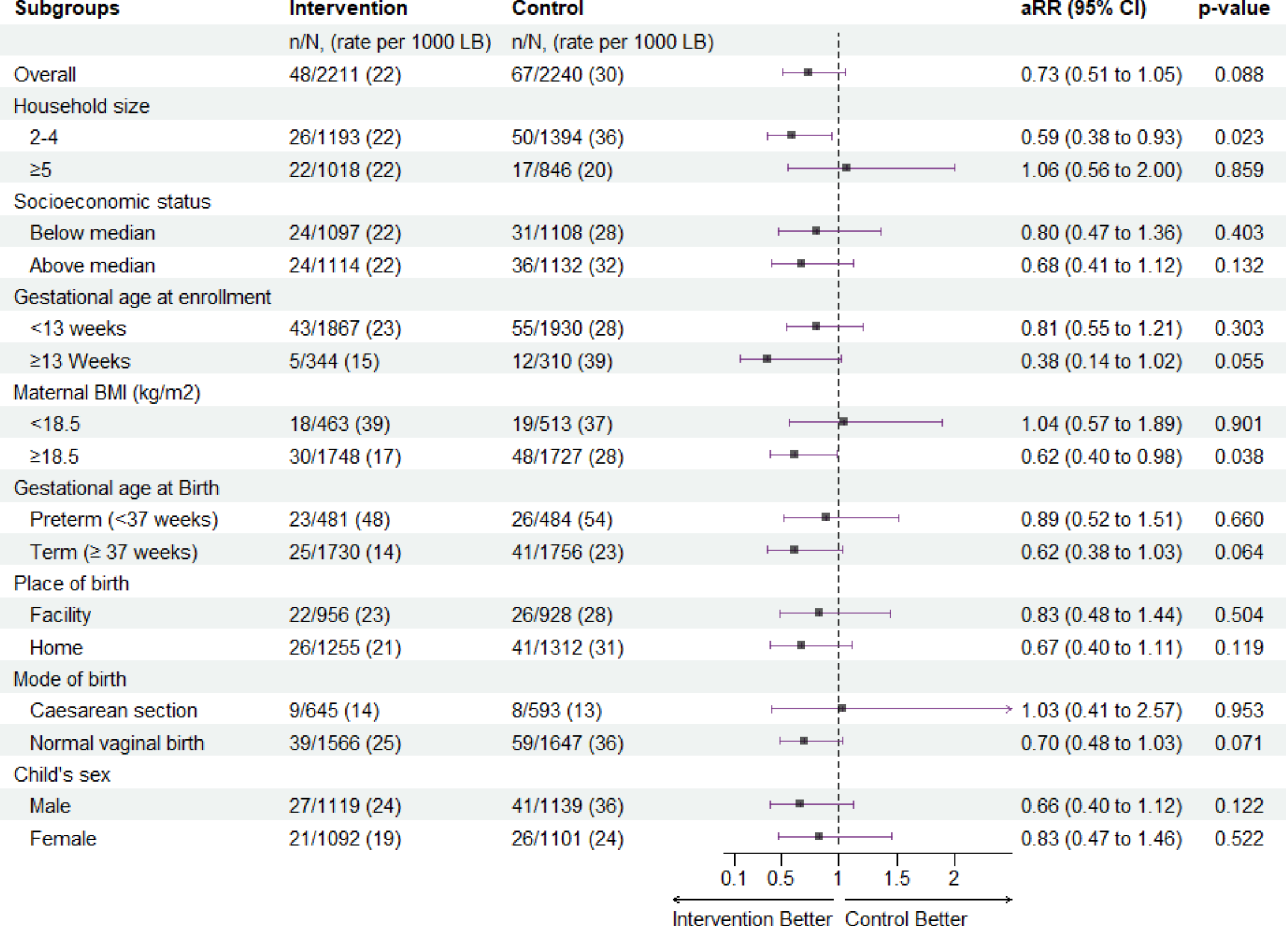
Effect of cooking with LPG on early neonatal mortality by subgroups, Poriborton trial. LB, livebirths, aRR, adjusted relative risk, adjusted for household size and maternal age. LPG, liquefied petroleum gas.

In post hoc subgroup analyses, the perinatal mortality rate was lower in the smaller sized households in the intervention group (67 deaths, rate 54 per 1000 births) than in the control group (102 deaths, 71 per 1000 births, adjusted RR (aRR) 0.75; 95% CI 0.56 to 1.00; p=0.047) (figure 2). The effect was larger in these smaller households, when we conducted the analyses in early neonatal deaths, with 26 deaths (22 per 1000 live births) in the intervention group compared with 50 deaths (36 per 1000 live births) (aRR 0.59; 95% CI (0.38 to 0.93; p=0.022) (figure 3). Further examination by subgroups demonstrated reduced risk estimates for perinatal mortality among women with BMI ≥18.5, term births and male infants with the effect favouring the intervention (figure 2). When we examined these subgroups among the early neonatal deaths, the results were similar, favouring the intervention in women with a BMI ≥18, vaginal births and term infants (figure 3). There was no difference between baseline characteristics based on household size (online supplemental table S4).

In the intervention group, exclusive LPG cooking practices differed by household size. With exclusive self-reported LPG use higher in smaller households (1201 of 1221 households (98.4%)) compared with larger households (956 of 1008 households (94.8%)). This persisted at the final follow-up, 1002 of 1076 (93.1%) for smaller households compared with 770 of 880 (87.5%) for larger households (online supplemental table S5). There was, however, no difference in measurements of PM_2.5_ exposure by household size, which was only available for a small number of households (online supplemental table S6).

There were two reports of adverse events; one house fire attributed to incorrect gas cylinder installation, and a 14-year child who received a minor burn while incorrectly using the gas cylinder.

## Discussion

In this community-based cluster RCT, we found that the perinatal mortality rate was substantially lower in households that used LPG for cooking compared with households that continued with usual cooking practices; however, the CI included the null value. When we considered other mortality outcomes, we found larger reductions in early neonatal mortality in intervention compared with control households, particularly in smaller households. These are important findings with a very large impact on infant mortality that strongly support adoption of clean cooking in settings where infant mortality is high, and there is low penetration of clean cooking fuels.

The strengths of our study include the cluster design with geographical buffers between the clusters, thus reducing the risk of LPG adoption in the control group, a single-site heterogeneous study location, separate teams for intervention and data collection who were blinded to the study hypothesis, blinded statistical analysis and high adherence to the protocol and follow-up. The efficacy of the intervention is evidenced by the reduction in PM_2.5_ demonstrating that adopting cleaner fuel reduces personal exposure, although not reaching the WHO IT-2 target. Although we did not assess adherence through stove use monitors, our monthly stove use monitoring visits data supported good adherence. The COVID lockdowns impacted our field work, including kitchen observation data, which was not available for the whole sample. Furthermore, this high-value intervention encouraged women in the intervention group to report suspected pregnancies at very early gestations. Consequently, pregnancy loss before 28 weeks gestation was higher in the intervention group compared with the control group. Pregnancy loss prior to 28 weeks gestation was an a priori exclusion; however, this may limit generalisability. Another limitation was our inability to assess the timing of stillbirth. The causes of stillbirth before birth and those during birth are different and having this data could have provided valuable insights.^3^

We consider this promising evidence of a reduction in neonatal outcomes associated with clean cooking; however, the inclusion of the null value in the CIs certainly makes the results open to other interpretations. Our interpretation is based on consideration of the balance of the entirety of the findings. First, we conclude that the sample size was insufficient to detect the reduction in perinatal mortality that we found compared with the larger effect that we proposed in the protocol. Our final sample size was based on several factors including the effect estimates from the systematic reviews, the perinatal mortality prevalence data from previous research in the study area and feasibility considering the increasing cost of LPG when we were finalising the protocol.^10 11 26^ The subgroup analyses strongly support the overall benefit of clean cooking, with smaller households having a large reduction in perinatal and early neonatal mortality. These results demonstrate differences in exclusive LPG fuel cooking practices between small and large households. We contest that smaller households’ cooking needs were adequately managed with the two-burner LPG stove and did not require more cooking stoves or fuels to meet their family meal requirements. In larger households, the two-burner cookstove did not meet the needs of the entire household, and there was likely other cooking using polluting fuel, thus exposing our participants to HAP. Our previous research supports this, in the feasibility study, we reported fuel saving practices and cooking challenges for large families on the small stove that we tested,^25^ and in our observational study among pregnant women who self-adopted LPG cooking, we reported higher LPG stove use (defined as 50% of their total cooking time in the preceding 24 hours) in smaller households than in larger households.^19^ We used these findings in the design of the Poriborton trial and our intervention included a larger stove that could accommodate larger cooking pots and unlimited LPG supply to support exclusive LPG cooking. However, we now consider that these solutions failed in exclusive LPG cooking for larger households. To the best of our knowledge and before we commenced the trial, there have been no other published reports on the effect of cooking practices or exposure associated with household size and hence, consider the association with perinatal mortality and household size a novel and important finding. Since we conducted the trial, there have been several reports of differences in fuel use and household or family size, thus supporting our finding.^27 28^ In the study setting, families live in tin structures called a homestead that are clustered together in proximity and include multigenerational members to form a household. Although we encouraged all household members to participate in the behaviour change communication sessions and to adopt clean cooking or to protect pregnant women from household cooking fumes, this may not have always occurred. In close and crowded homesteads, our individual intervention may have been insufficient to reduce personal exposure for the duration of pregnancy in larger households. Our finding of a differential effect in households of smaller size compared with larger size provides evidence of the need to provide access to clean fuel at the community level. This is supported by an expert meeting unpacking the null results of three high-quality randomised controlled trials of water sanitation and hygiene interventions to improve childhood stunting.^29^ The consensus was that interventions delivered at the household level could not affect changes in childhood stunting, which is a multifactorial chronic condition and called for more ambitious community-level interventions that combine multiple sectors, infrastructure and behaviour. This is very relevant for HAP and perinatal morbidity and mortality.

Furthermore, the null effect on perinatal mortality, which is a composite measure of stillbirth and early neonatal mortality, appears to be driven by a lack of an effect on stillbirth. This is likely due to differences in the causes of stillbirth and early neonatal deaths. Stillbirth is high in northern Bangladesh, and the causes of stillbirth are highly prevalent, including inadequate antenatal and perinatal care. The protective effect from clean cooking may be insufficient to negate these prevalent risk factors, all of which are pathways to stillbirth.^30 31^ For infants who survive pregnancy and birth, being born into a relatively cleaner air environment may have been protective against respiratory morbidity, which is a major cause of early neonatal mortality. The effect in the subgroup analysis further supports this, as the intervention was more effective in infants whose mother did not have risk factors requiring obstetric intervention (eg, had a normal BMI, had a vaginal birth and did not attend a health facility).^32^

In this setting where home birth is the preferred setting, facility births are often an indicator of a transfer to that setting due to high-risk pregnancy or a complication during the birth and may be associated with delayed referral and therefore increased risk due to delayed health seeking, such as the Three Delay Model, and our own previous work.^33 34^

The PM_2.5_ exposure was impacted by the ambient seasonal conditions. The second assessment occurred during the monsoon season, whereas the third assessment occurred during the dry season. The heavy rain during the second assessment depressed the ambient concentrations, whereas during the third visit, the lack of rain increased ambient PM_2.5_ concentrations and the corresponding personal PM_2.5_ concentrations. This is consistent with previous research conducted in Bangladesh.^35^,^37^ It should be noted that these assessments were conducted in a small subset and not the whole study sample. The reduction in exposure of up to 29% aligns with the Ghana Randomized Air Pollution and Health Study (GRAPHS), which achieved a 32% reduction, but not with the difference reported in the HAPIN trial, which achieved up to 77% reduction.^38 39^ These reductions demonstrate that LPG may be useful as a transitional fuel in settings where biomass is still the main fuel.

Poriborton is the largest of the RCT’s investigating the impact of clean cooking interventions on maternal and neonatal outcomes, and together with HAPIN and GRAPHS, all have yielded mixed but informative results.^16 38^ The HAPIN trial, conducted across four countries, demonstrated substantial reductions in personal exposure but did not find significant effects on birth weight or perinatal mortality, and the country-level results are heterogeneous. Similarly, the GRAPHS trial in Ghana also reduced personal exposure and improved infant growth trajectories. These findings suggest that while clean cooking interventions can reduce exposure to HAP, their impact on pregnancy outcomes is not clear. We propose to conduct together with other trial lists an individual participant data meta-analysis. This individual-level data will increase power and enable more precise estimation of intervention effects, explore subgroups and identify effect modifiers to better understand exposure–outcome relationships.

Our findings are important and provide promising evidence that the use of LPG for cooking can reduce early neonatal mortality, particularly in smaller households. We found novel evidence of a reduction in early neonatal mortality due to reduced exposure to PM_2.5_, especially in smaller households, which reported high levels of adherence with exclusive LPG cooking. Similar to other complex and multifactorial morbidity outcomes, we recommend that a community-level intervention that addresses clean fuel infrastructure will lead to improvements in infant survival in settings where biomass fuel remains the dominant fuel.

## Supplementary material

10.1136/bmjgh-2025-020391 online supplemental figure 1

10.1136/bmjgh-2025-020391 online supplemental figure 2

10.1136/bmjgh-2025-020391 online supplemental table 1

10.1136/bmjgh-2025-020391 online supplemental file 1

10.1136/bmjgh-2025-020391 online supplemental file 2

10.1136/bmjgh-2025-020391 online supplemental file 3

## Data Availability

Data are available upon reasonable request.
