# Supplementary figures and images for: Impact of cooking with liquefied petroleum gas compared with traditional cooking practices on perinatal and early neonatal mortality: the Poriborton cluster randomised controlled trial
Source: BMJ Glob Health. 2026 Feb 16;11(2):e020391. doi: 10.1136/bmjgh-2025-020391 (PMC12911768; doi:10.1136/bmjgh-2025-020391)

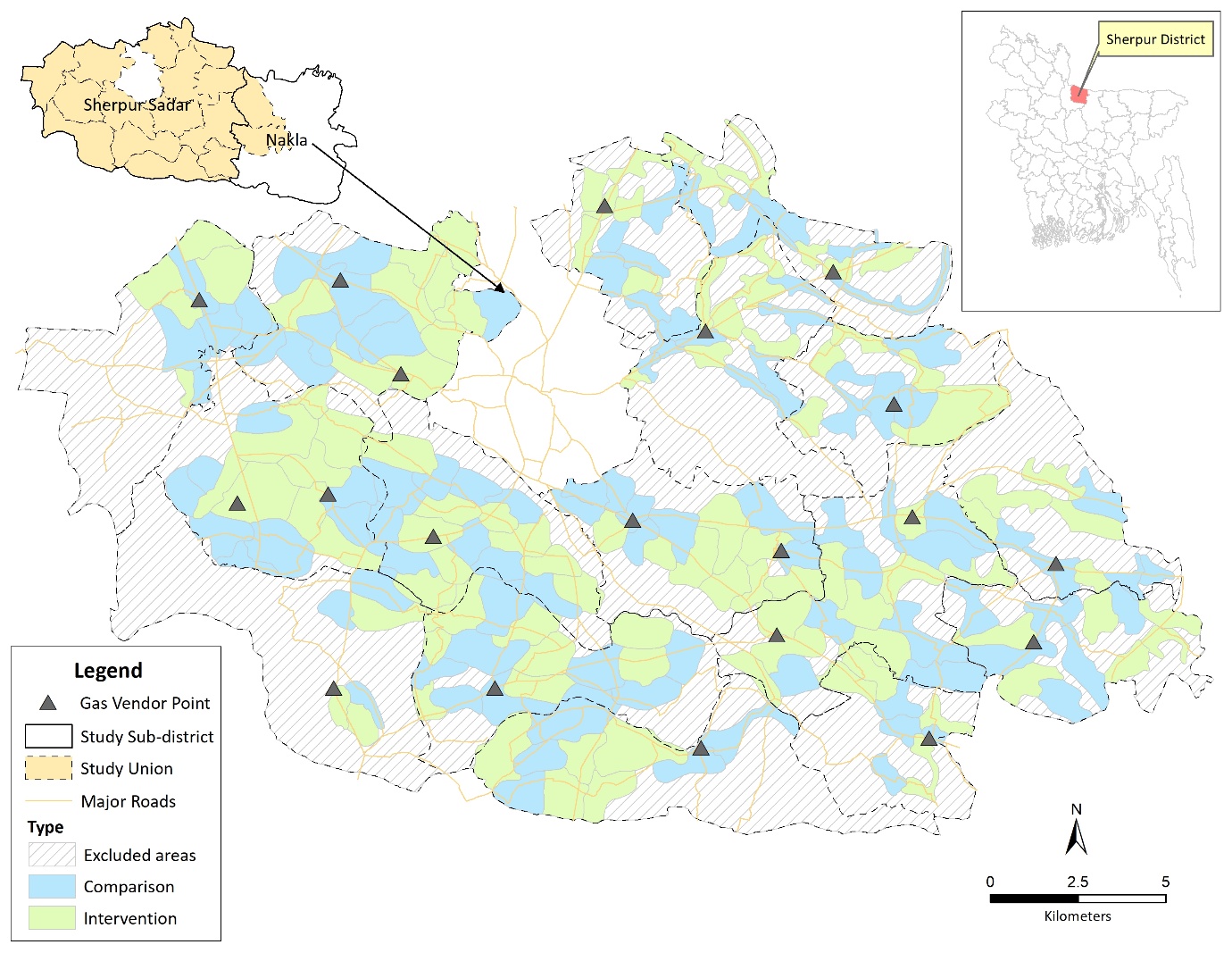


**Supplemental Figure 1:Map of Poriborton trial study site and clusters.**

Supplement: online supplemental figure 1 [file bmjgh-11-2-s001.docx]

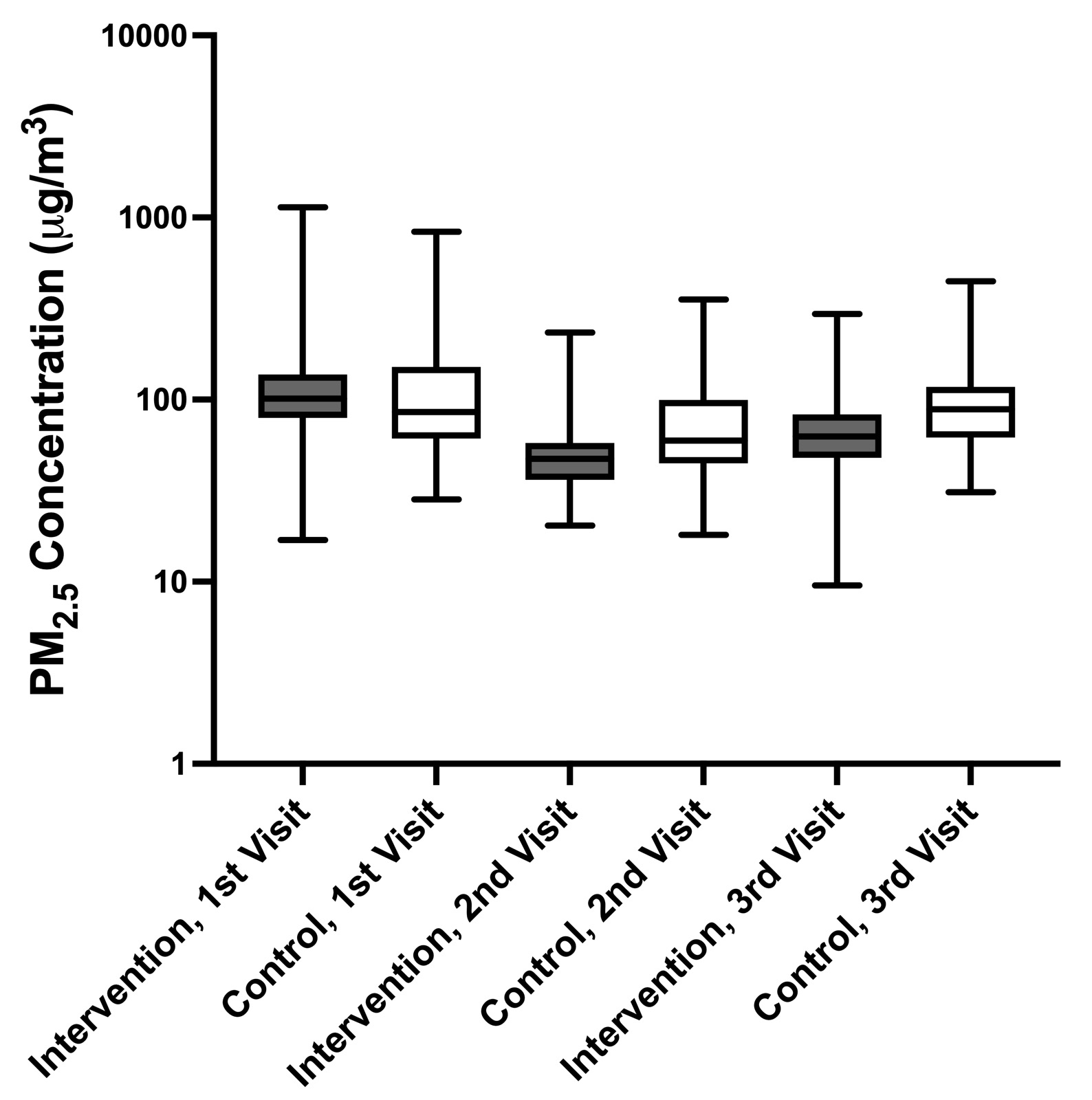

Supplement: online supplemental figure 2 [file bmjgh-11-2-s002.jpg]
