# Supplementary material for: Impact of cooking with liquefied petroleum gas compared with traditional cooking practices on perinatal and early neonatal mortality: the Poriborton cluster randomised controlled trial
Source: BMJ Glob Health. 2026 Feb 16;11(2):e020391. doi: 10.1136/bmjgh-2025-020391 (PMC12911768; doi:10.1136/bmjgh-2025-020391)
Supplement: online supplemental table 1 [file bmjgh-11-2-s003.docx]

**Supplement Table S1: Birth characteristics among total births, Poriborton trial.**

| **Characteristic** | **Intervention (n=2278)** | **Control (n=2314)** |
| --- | --- | --- |
| Place of birth —n (%) |  |  |
| Health facility | 987 (43.3) | 954 (41.2) |
| Home or other non-health facility | 1291 (56.7) | 1360 (58.8) |
| Mode of birth—n (%) |  |  |
| Vaginal birth | 1623 (71.2) | 1710 (73.9) |
| Caesarean section | 655 (28.8) | 604 (26.1) |
| Type of birth—n (%) |  |  |
| Singleton | 2246 (98.6) | 2280 (98.5) |
| Multiple | 32 (1.4) | 34 (1.5) |

**Supplement Table S2: Self-reported fuel use in previous 24hr, at three time points, Poriborton trial**

| **Type of fuel used** | **Enrolment - baseline (pre intervention)** | | **1^st^ follow-up(5-6 months)** | | **2nd follow-up (before the birth)** | |
| --- | --- | --- | --- | --- | --- | --- |
|  | **Intervention**  **n (%)** | **Control**  **n (%)** | **Intervention**  **n (%)** | **Control**  **n (%)** | **Intervention**  **n (%)** | **Control**  **n (%)** |
| N | 2472 | 2472 | 2270 | 2304 | 1987 | 2097 |
| Biomass fuel only | 2123 (85.9) | 2016 (81.6) | 20 (0.9) | 1863 (80.9) | 106 (5.3) | 1635 (78.0) |
| Cleaner fuel only (biogas, electricity, LPG) | 37 (1.5) | 40 (1.6) | 2198 (96.8) | 64 (2.8) | 1803 (90.7) | 121 (5.8) |
| Mixed (biomass and cleaner) | 312 (12.6) | 416 (16.8) | 52 (2.3) | 377 (16.4) | 78 (3.9) | 341 (16.3) |

**Supplement Table S3: Personal exposure to Particulate Matter 2.5, Poriborton Trial**

|  |  | Intervention |  | Control |  |  |
| --- | --- | --- | --- | --- | --- | --- |
| Exposure visit  [mean±SD gestational age in months] | n | Mean (SD)  Median (IQR) | n | Mean (SD)  Median (IQR) | Mean Difference  Intervention-control (95%CI) | P-value |
| First [4.3±0.9 m] | 151 | 122.2 (101.0)  101.4 (57.5) | 152 | 123.1 (104.0)  83.5 (89.3) | -0.005 (-0.003, 0.000)* | 0.133 |
|  |  |  |  |  |  |  |
| Second [6.4±0.6 m] | 119 | 56.3 (37.0)  47.2 (20.9) | 119 | 82.1 (59.6)  59.6 (53.1) | -0.133 (-0.194, -0.072)† | <0.001 |
|  |  |  |  |  |  |  |
| Third  [8.0±0.6 m] | 130 | 70.3 (35.7)  62.7 (34.5) | 134 | 101.1 (57.1)  88.7 (54.3) | -0.149 (-0.198, -0.101) | <0.001 |
|  |  |  |  |  |  |  |

**Supplement Table S4: Distribution of background characterises by household size, small versus large, Poriborton Trial**

|  | **Small Households (size** 2-4) | | | **Large Households (size** ≥5) | | |
| --- | --- | --- | --- | --- | --- | --- |
| **Baseline characteristics** | **Intervention**  **(N=1344)** | **Control**  **(N=1544)** | **p-value** | **Intervention**  **(N=1128)** | **Control**  **(N=928)** | **p-value** |
|  |  |  |  |  |  |  |
| Woman's age (years) —n (%) |  |  |  |  |  |  |
| < 20 | 252 (18.8) | 243 (15.7) | 0.126 | 291 (25.8) | 222 (23.9) | 0.56 |
| 20-24 | 417 (31.0) | 504 (32.6) |  | 340 (30.1) | 283 (30.5) |  |
| 25-29 | 422 (31.4) | 523 (33.9) |  | 235 (20.8) | 216 (23.3) |  |
| >30 | 253 (18.8) | 274 (17.7) |  | 262 (23.2) | 207 (22.3) |  |
|  |  |  |  |  |  |  |
| Maternal BMI (kg/m2) —n (%) |  |  |  |  |  |  |
| <18.5 | 314 (23.4) | 367 (23.8) | 0.802 | 208 (18.4) | 199 (21.4) | 0.105 |
| ≥18.5 | 1030 (76.6) | 1177 (76.2) |  | 920 (81.6) | 729 (78.6) |  |
|  |  |  |  |  |  |  |
| Gestational age at enrolment (weeks) —n (%) |  |  |  |  |  |  |
| ≤ 12 weeks | 1160 (86.4) | 1348 (87.3) |  | 958 (84.9) | 801 (86.3) |  |
| > 12 weeks | 184 (13.7) | 196 (12.7) | 0.463 | 170 (15.1) | 127 (13.7) | 0.377 |
|  |  |  |  |  |  |  |
| Parity |  |  |  |  |  |  |
| None | 375 (27.9) | 382 (24.7) |  | 450 (39.9) | 357 (38.5) |  |
| 1 | 482 (35.9) | 586 (38.0) | 0.189 | 290 (25.7) | 214 (23.1) | 0.180 |
| 2 or more | 487 (36.2) | 576 (37.3) |  | 388 (34.4) | 357 (38.5) |  |
|  |  |  |  |  |  |  |
| Household wealth quintiles —n (%) |  |  |  |  |  |  |
| Lowest (poorest) | 340 (25.3) | 373 (24.2) |  | 142 (12.6) | 134 (14.4) |  |
| Second | 321 (23.9) | 337 (21.8) | 0.566 | 186 (16.5) | 145 (15.6) | 0.207 |
| Middle | 266 (19.8) | 335 (21.7) |  | 223 (19.8) | 165 (17.8) |  |
| Fourth | 214 (15.9) | 260 (16.8) |  | 299 (26.5) | 217 (23.4) |  |
| Highest | 203 (15.1) | 239 (15.5) |  | 278 (24.6) | 267 (28.8) |  |

**Supplement Table S5: Exclusive LPG use by kitchen observation in intervention group by household size, Poriborton Trial.**

| Timepoint | Large households | | Small households | | RR (95% CI) | p-value |
| --- | --- | --- | --- | --- | --- | --- |
|  | N | n (%) | N | n (%) |  |  |
| At enrolment | 1127 | 10 (0.9) | 1334 | 16 (1.2) | 0.74 (0.34 to 1.61) | 0.45 |
| 1^st^ follow-up | 1008 | 956 (94.8) | 1221 | 1201 (98.4) | 0.96 (0.94 to 0.99) | 0.001 |
| 2^nd^ follow-up | 880 | 770 (87.5) | 1076 | 1002 (93.1) | 0.94 (0.91 to 0.97) | <0.001 |

**Supplement Table S6:PM_2.5_ concentrations differences between household sizes within the intervention group, Poriborton Trial.**

| **Intervention group** |  | **HH ≤ 4** |  | **HH ≥ 5** |  |
| --- | --- | --- | --- | --- | --- |
| Follow-up timepoint | n | Mean (SD)  Median (IQR) | n | Mean (SD)  Median (IQR) | P-value |
| First | 67 | 112.1 (54.1)  99.9 (61.9) | 84 | 130.3 (126.4)  101.6 (55.0) | 0.227 |
|  |  |  |  |  |  |
| Second | 55 | 55.9 (36.7)  47.8 (24.2) | 64 | 56.6 (37.5)  46.7 (19.4) | 0.996 |
|  |  |  |  |  |  |
| Third | 59 | 70.5 (42.9)  59.6 (33.1) | 71 | 70.1 (28.6)  63.4 (37.3) | 0.750 |
|  |  |  |  |  |  |
