# Supplementary material for: Impact of cooking with liquefied petroleum gas compared with traditional cooking practices on perinatal and early neonatal mortality: the Poriborton cluster randomised controlled trial
Source: BMJ Glob Health. 2026 Feb 16;11(2):e020391. doi: 10.1136/bmjgh-2025-020391 (PMC12911768; doi:10.1136/bmjgh-2025-020391)
Supplement: online supplemental file 1 [file bmjgh-11-2-s004.pdf]

গ্যাসের চুলায় একই সময়ে রান্না করার পাশাপাশি সবজি কাটা যায়

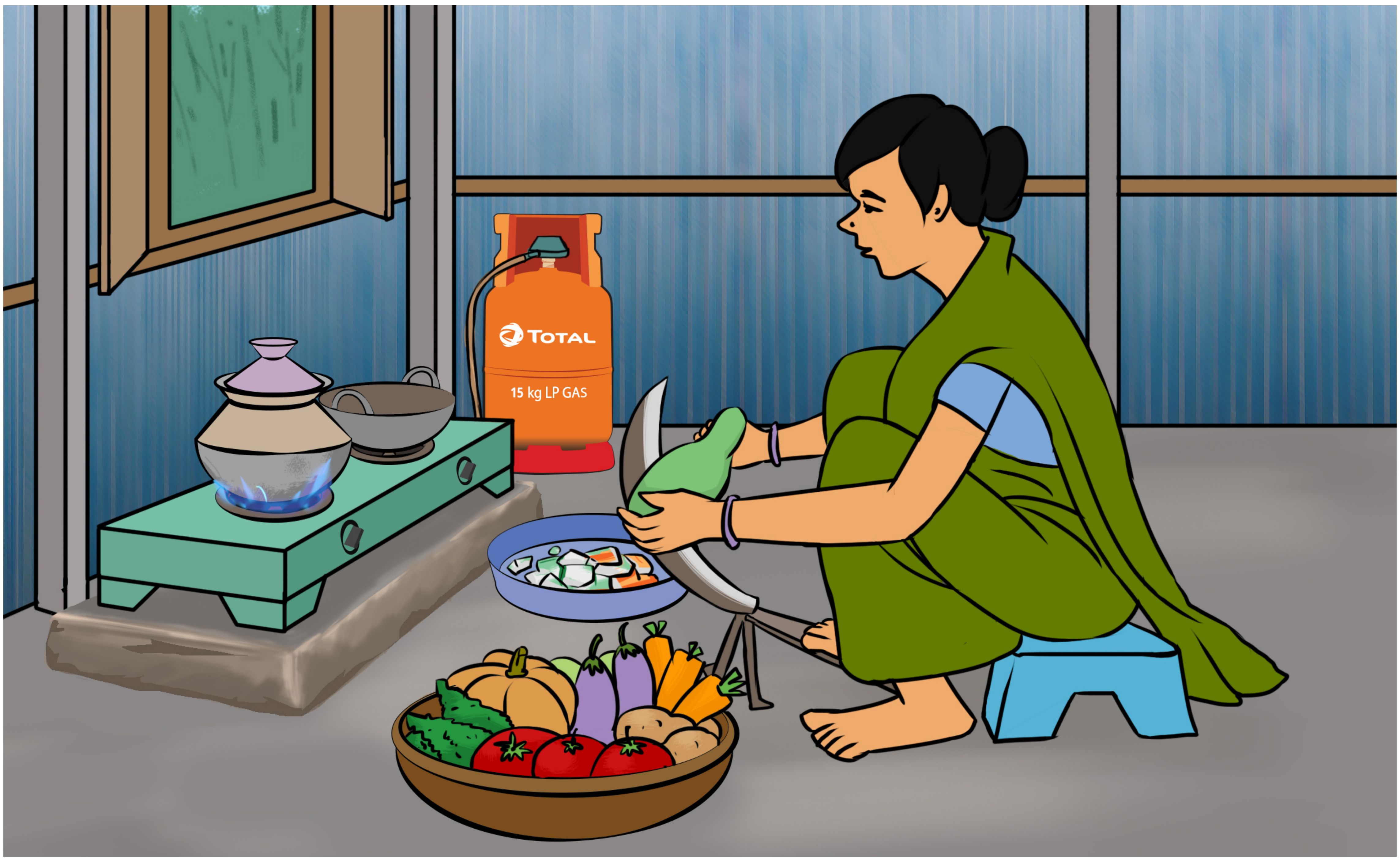

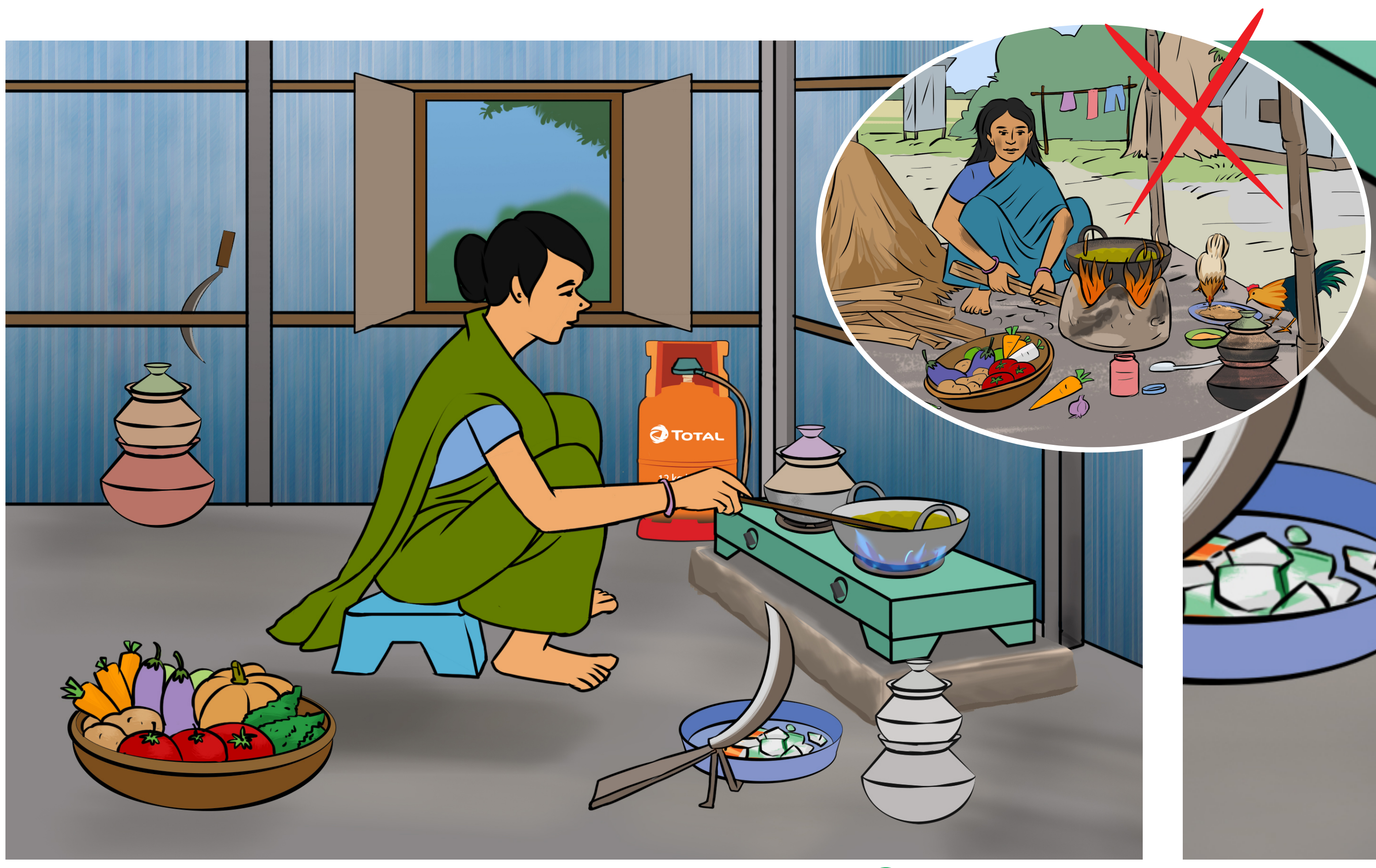

গ্যাসের চুলা ব্যবহারে কাপড়, চুলা এবং শরীর ময়লা ও দুর্গন্ধ হবে না

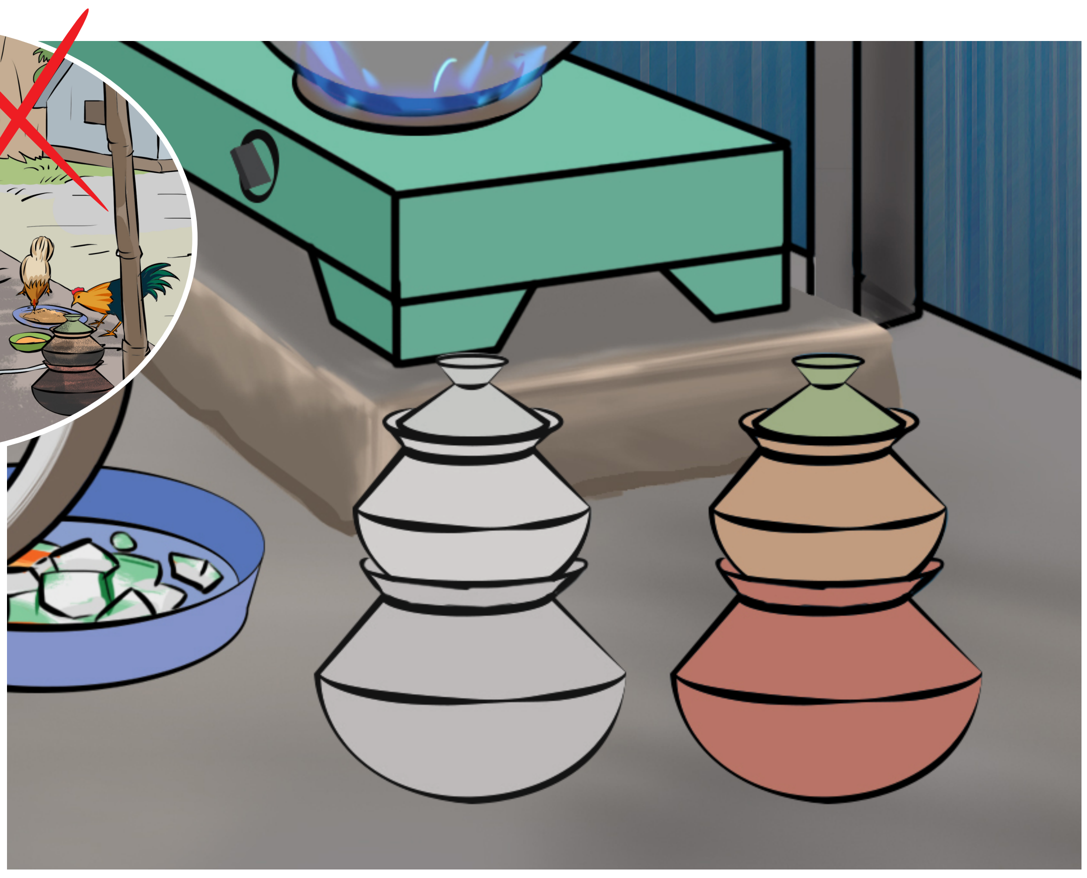

গ্যাসের চুলায় আপনার হাড়ি পাতিল কম ময়লা হবে

২০১৯

মে

রবি	সোম	মঙ্গল	বুধ	বৃহঃ	শুক্র	শনি
			০১	০২	০৩	০৪
০৫	০৬	০৭	০৮	০৯	১০	১১
১২	১৩	১৪	১৫	১৬	১৭	১৮
১৯	২০	২১	২২	২৩	২৪	২৫
২৬	২৭	২৮	২৯	৩০	৩১	

জুন

রবি	সোম	মঙ্গল	বুধ	বৃহঃ	শুক্র	শনি
৩০						০১
০২	০৩	০৪	০৫	০৬	০৭	০৮
০৯	১০	১১	১২	১৩	১৪	১৫
১৬	১৭	১৮	১৯	২০	২১	২২
২৩	২৪	২৫	২৬	২৭	২৮	২৯

জুলাই

রবি	সোম	মঙ্গল	বুধ	বৃহঃ	শুক্র	শনি
	০১	০২	০৩	০৪	০৫	০৬
০৭	০৮	০৯	১০	১১	১২	১৩
১৪	১৫	১৬	১৭	১৮	১৯	২০
২১	২২	২৩	২৪	২৫	২৬	২৭
২৮	২৯	৩০	৩১			

PORIBORTON
THE CHANGE TRIAL

THE UNIVERSITY OF SYDNEY

icddr,b

TOTAL

বিশেষ এবং পারিবারিক অনুষ্ঠানসহ আপনার পত্যেক বেলার রান্না অবশ্যই গ্যাসের চুলায় করবেন

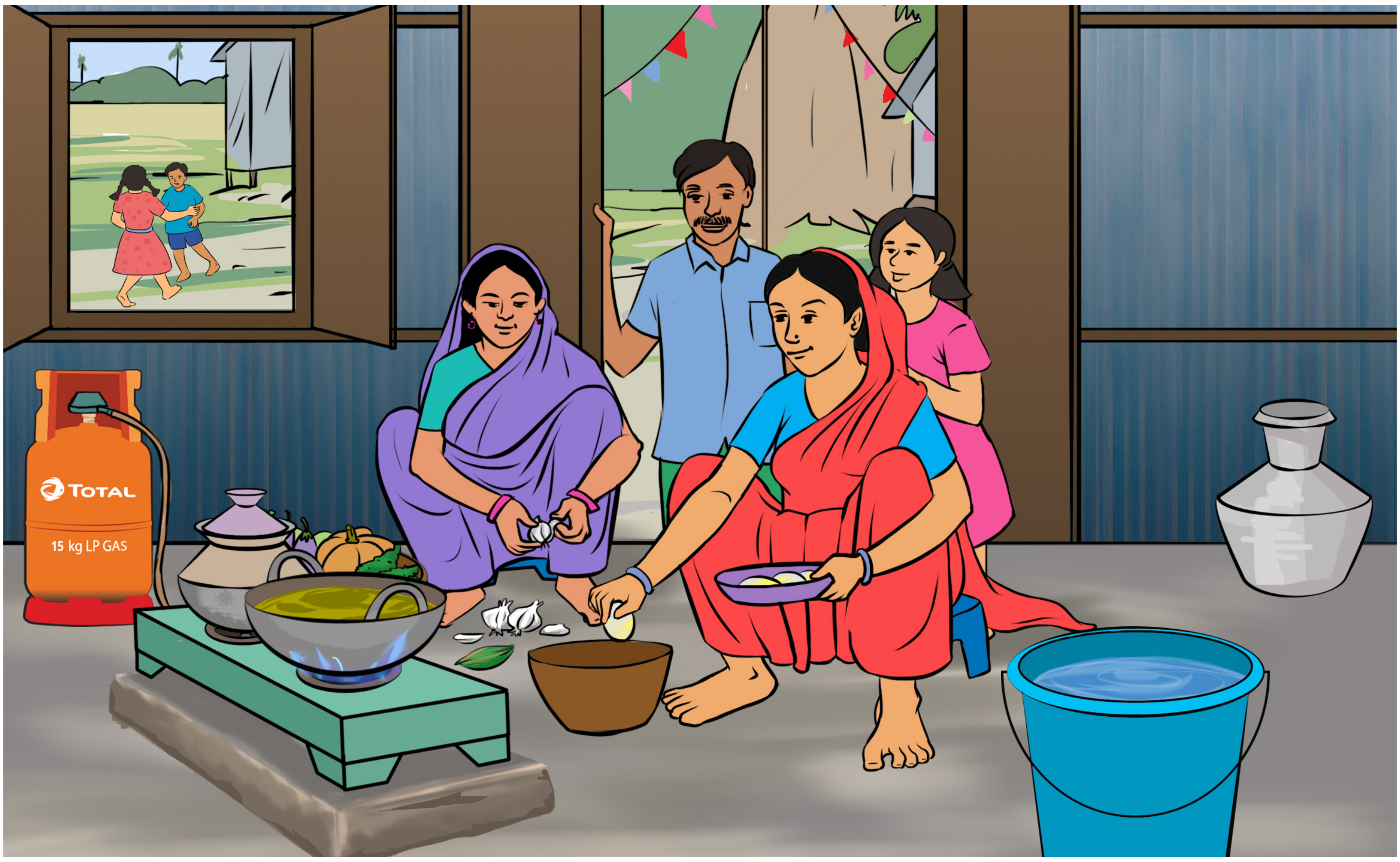

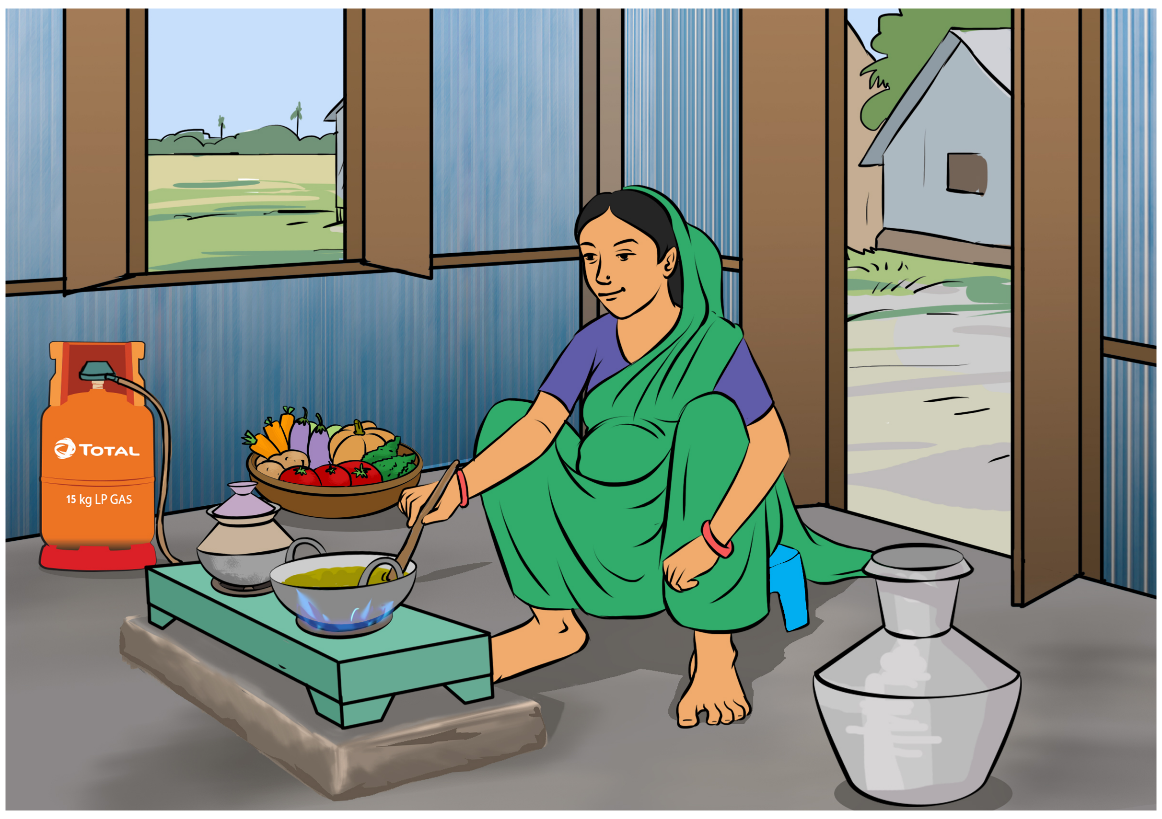

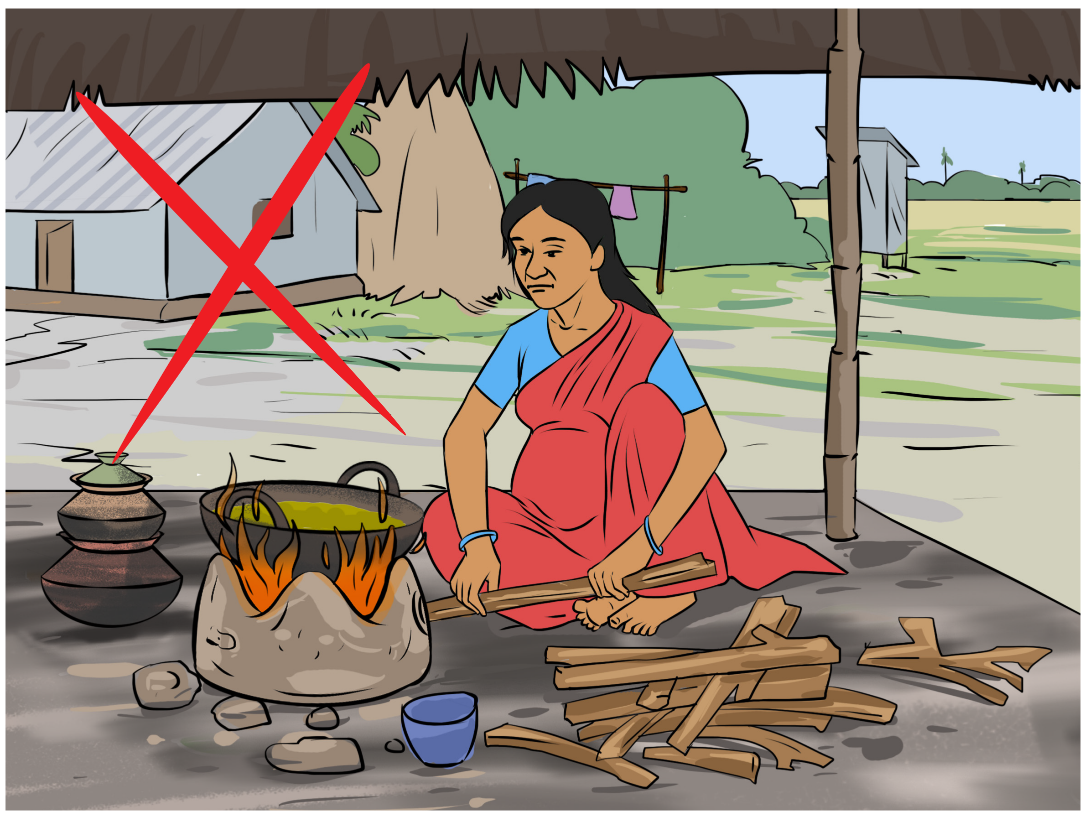

মনে রাখবেন, গর্ভকালীন সময়ে কোন কাজেই গ্যাসের চুলা ব্যতীত অন্য কোন চুলা ব্যবহার করবেন না

২০১৯

আগস্ট

রবি	সোম	মঙ্গল	বুধ	বৃহঃ	শুক্র	শনি
				০১	০২	০৩
০৪	০৫	০৬	০৭	০৮	০৯	১০
১১	১২	১৩	১৪	১৫	১৬	১৭
১৮	১৯	২০	২১	২২	২৩	২৪
২৫	২৬	২৭	২৮	২৯	৩০	৩১

সেপ্টেম্বর

রবি	সোম	মঙ্গল	বুধ	বৃহঃ	শুক্র	শনি
০১	০২	০৩	০৪	০৫	০৬	০৭
০৮	০৯	১০	১১	১২	১৩	১৪
১৫	১৬	১৭	১৮	১৯	২০	২১
২২	২৩	২৪	২৫	২৬	২৭	২৮
২৯	৩০					

অক্টোবর

রবি	সোম	মঙ্গল	বুধ	বৃহঃ	শুক্র	শনি
		০১	০২	০৩	০৪	০৫
০৬	০৭	০৮	০৯	১০	১১	১২
১৩	১৪	১৫	১৬	১৭	১৮	১৯
২০	২১	২২	২৩	২৪	২৫	২৬
২৭	২৮	২৯	৩০	৩১		

PORIBORTON
THE CHANGE TRIAL

Organize by

THE UNIVERSITY OF SYDNEY

icddr,b

TOTAL

সবসময় গ্যাসের চুলা ব্যবহার করলে গর্ভের বাচ্চা সঠিকভাবে বেড়ে ওঠে এবং সুস্থ ভাবে জন্ম নেয়

খড়ির চুলায় রান্না করলে গর্ভের বাচ্চার ক্ষতি হয়

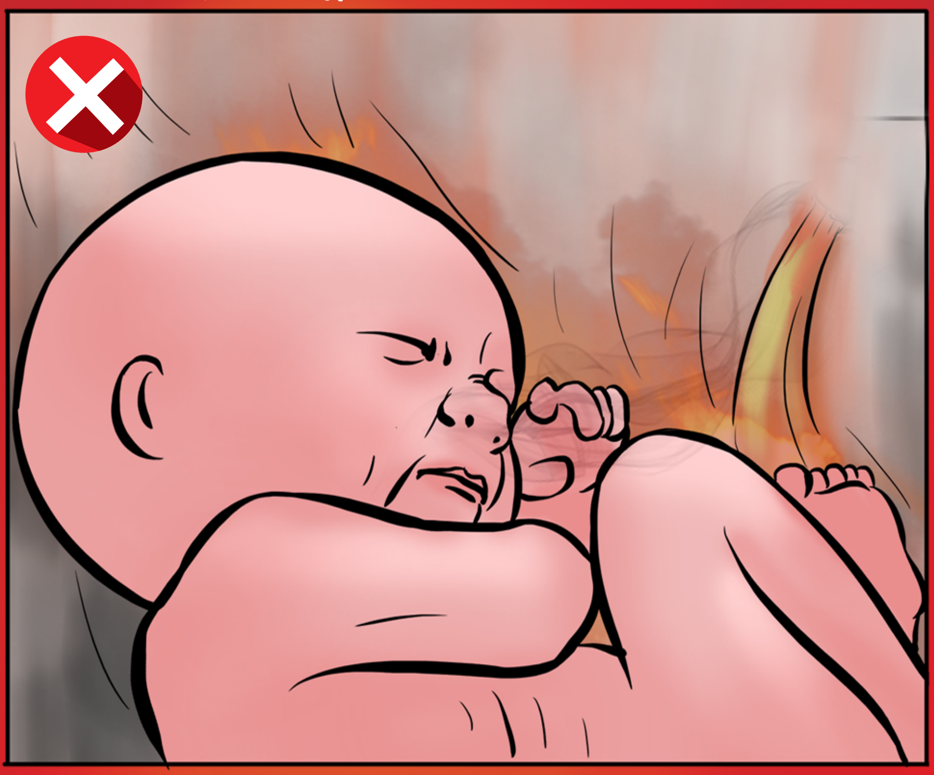

গ্যাসের চুলায় রান্না করলে গর্ভের বাচ্চার ক্ষতি হয় না

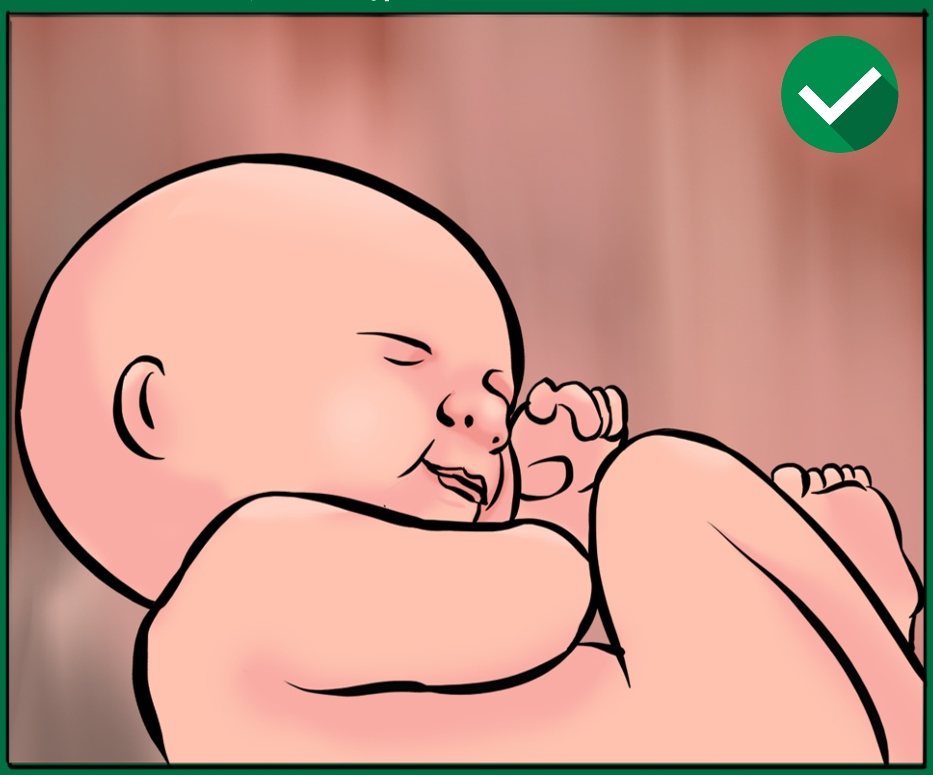

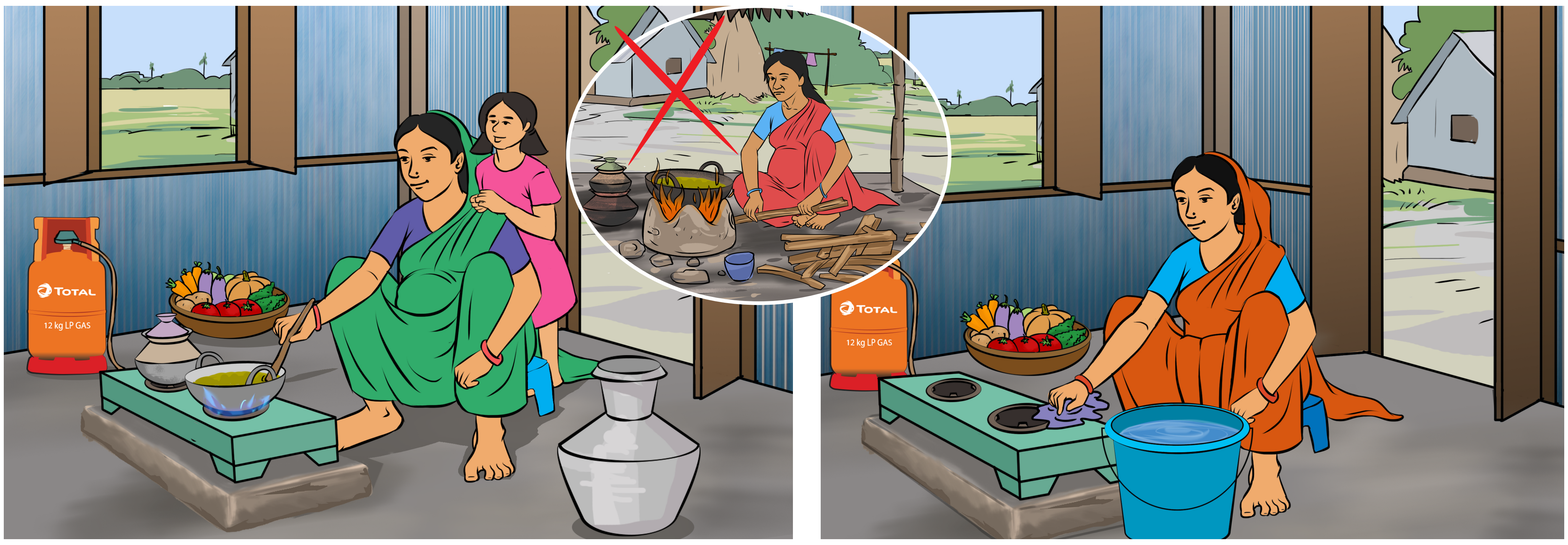

সবসময় গ্যাসের চুলা ব্যবহার করলে গর্ভের বাচ্চা সঠিকভাবে বেড়ে ওঠে এবং সুস্থ ভাবে জন্ম নেয়

গ্যাসের চুলা পরিষ্কারে আপনার অনেক কম সময় লাগবে

২০১৯-২০

নভেম্বর

রবি	সোম	মঙ্গল	বুধ	বৃহঃ	শুক্র	শনি
					০১	০২
০৩	০৪	০৫	০৬	০৭	০৮	০৯
১০	১১	১২	১৩	১৪	১৫	১৬
১৭	১৮	১৯	২০	২১	২২	২৩
২৪	২৫	২৬	২৭	২৮	২৯	৩০

ডিসেম্বর

রবি	সোম	মঙ্গল	বুধ	বৃহঃ	শুক্র	শনি
০১	০২	০৩	০৪	০৫	০৬	০৭
০৮	০৯	১০	১১	১২	১৩	১৪
১৫	১৬	১৭	১৮	১৯	২০	২১
২২	২৩	২৪	২৫	২৬	২৭	২৮
২৯	৩০	৩১				

জানুয়ারি

রবি	সোম	মঙ্গল	বুধ	বৃহঃ	শুক্র	শনি
			০১	০২	০৩	০৪
০৫	০৬	০৭	০৮	০৯	১০	১১
১২	১৩	১৪	১৫	১৬	১৭	১৮
১৯	২০	২১	২২	২৩	২৪	২৫
২৬	২৭	২৮	২৯	৩০	৩১	

PORIBORTON
THE CHANGE TRIAL

Organize by

THE UNIVERSITY OF SYDNEY

icddr,b

TOTAL

গ্যাসের চুলাটি ব্যাবহারের সময় ঘরের দরজা ও জানালা খোলা রাখুন।

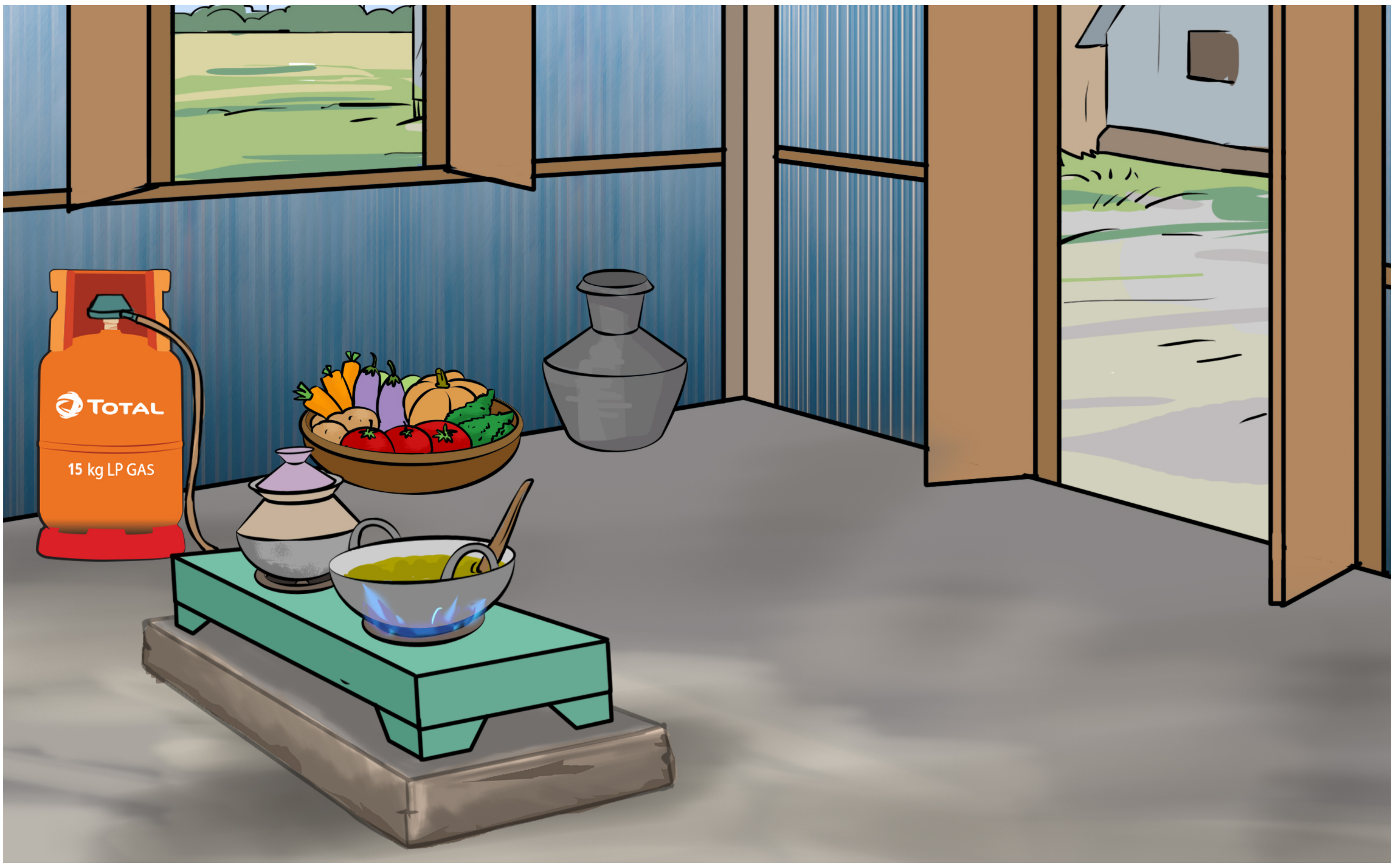

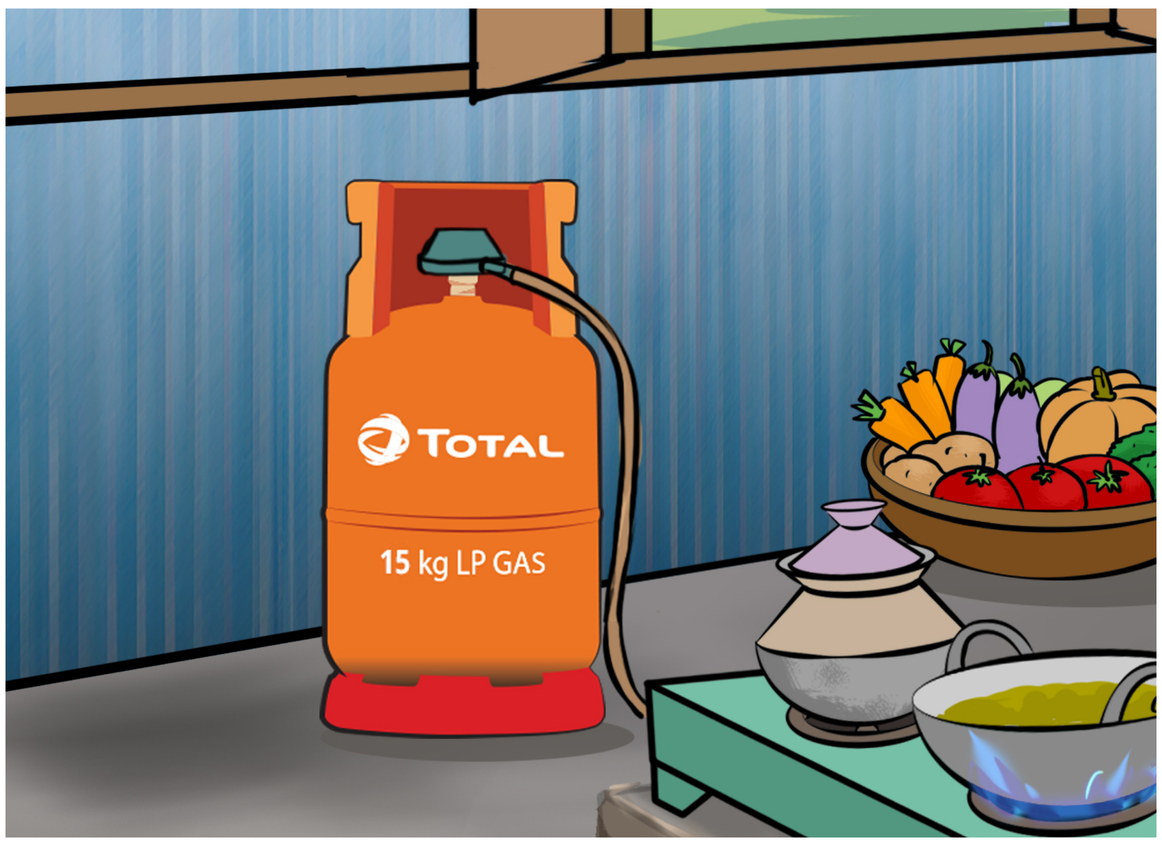

আপনার গ্যাস সিলিন্ডারটি অবশ্যই সবসময় লম্বালম্বি দাঁড় করিয়ে রাখুন

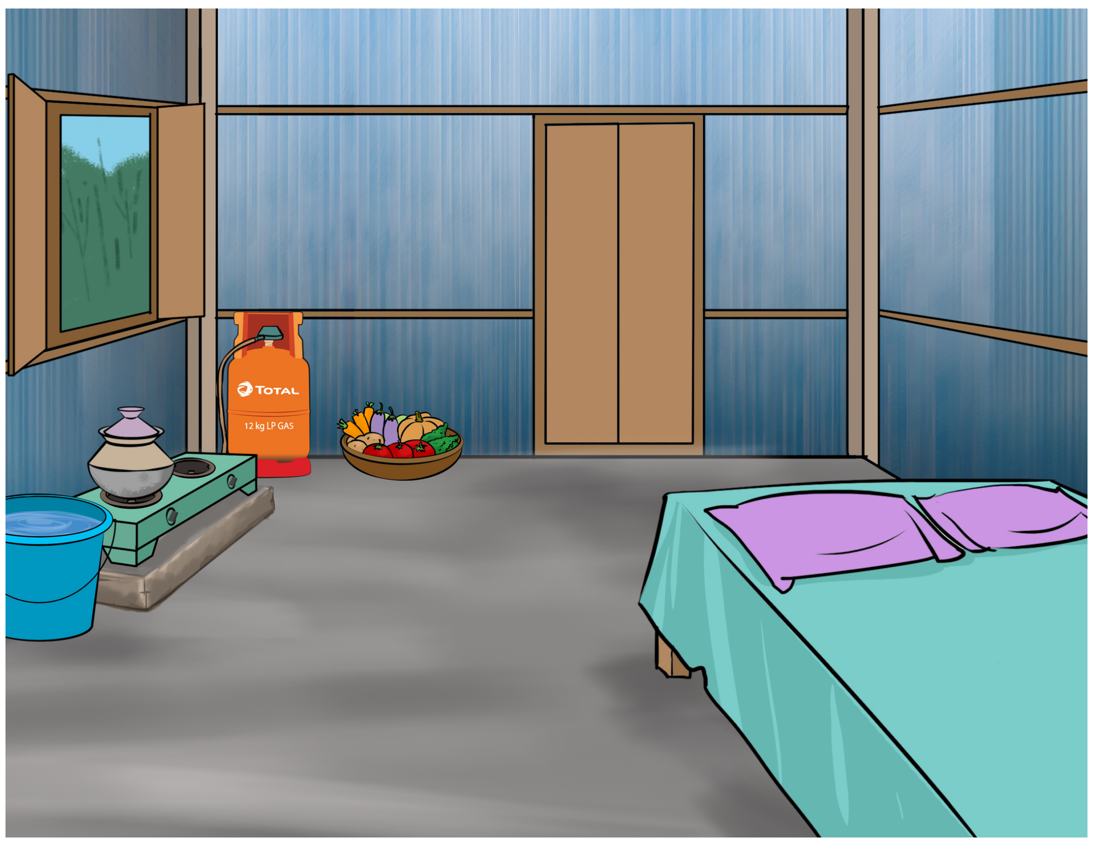

গ্যাসের চুলা ব্যবহারে ছাদ, দেয়াল এবং আসবাব ময়লা, নোংরা কিংবা নষ্ট হবে না

২০২০

ফেব্রুয়ারী

রবি	সোম	মঙ্গল	বুধ	বৃহঃ	শুক্র	শনি
						০১
০২	০৩	০৪	০৫	০৬	০৭	০৮
০৯	১০	১১	১২	১৩	১৪	১৫
১৬	১৭	১৮	১৯	২০	২১	২২
২৩	২৪	২৫	২৬	২৭	২৮	২৯

মার্চ

রবি	সোম	মঙ্গল	বুধ	বৃহঃ	শুক্র	শনি
০১	০২	০৩	০৪	০৫	০৬	০৭
০৮	০৯	১০	১১	১২	১৩	১৪
১৫	১৬	১৭	১৮	১৯	২০	২১
২২	২৩	২৪	২৫	২৬	২৭	২৮
২৯	৩০	৩১				

এপ্রিল

রবি	সোম	মঙ্গল	বুধ	বৃহঃ	শুক্র	শনি
			০১	০২	০৩	০৪
০৫	০৬	০৭	০৮	০৯	১০	১১
১২	১৩	১৪	১৫	১৬	১৭	১৮
১৯	২০	২১	২২	২৩	২৪	২৫
২৬	২৭	২৮	২৯	৩০		

PORIBORTON
The Change Trial

Organize by
THE UNIVERSITY OF SYDNEY
icddr,b
TOTAL
