# Supplementary material for: Impact of cooking with liquefied petroleum gas compared with traditional cooking practices on perinatal and early neonatal mortality: the Poriborton cluster randomised controlled trial
Source: BMJ Glob Health. 2026 Feb 16;11(2):e020391. doi: 10.1136/bmjgh-2025-020391 (PMC12911768; doi:10.1136/bmjgh-2025-020391)

PORIBORTON

THE CHANGE TRIAL

গ্যাসের চুলায় করলে রান্না মা বাচ্চা থাকবো ভালা

A HEALTHCARE PROGRAM BY

THE UNIVERSITY OF SYDNEY

icddr,b

TOTAL

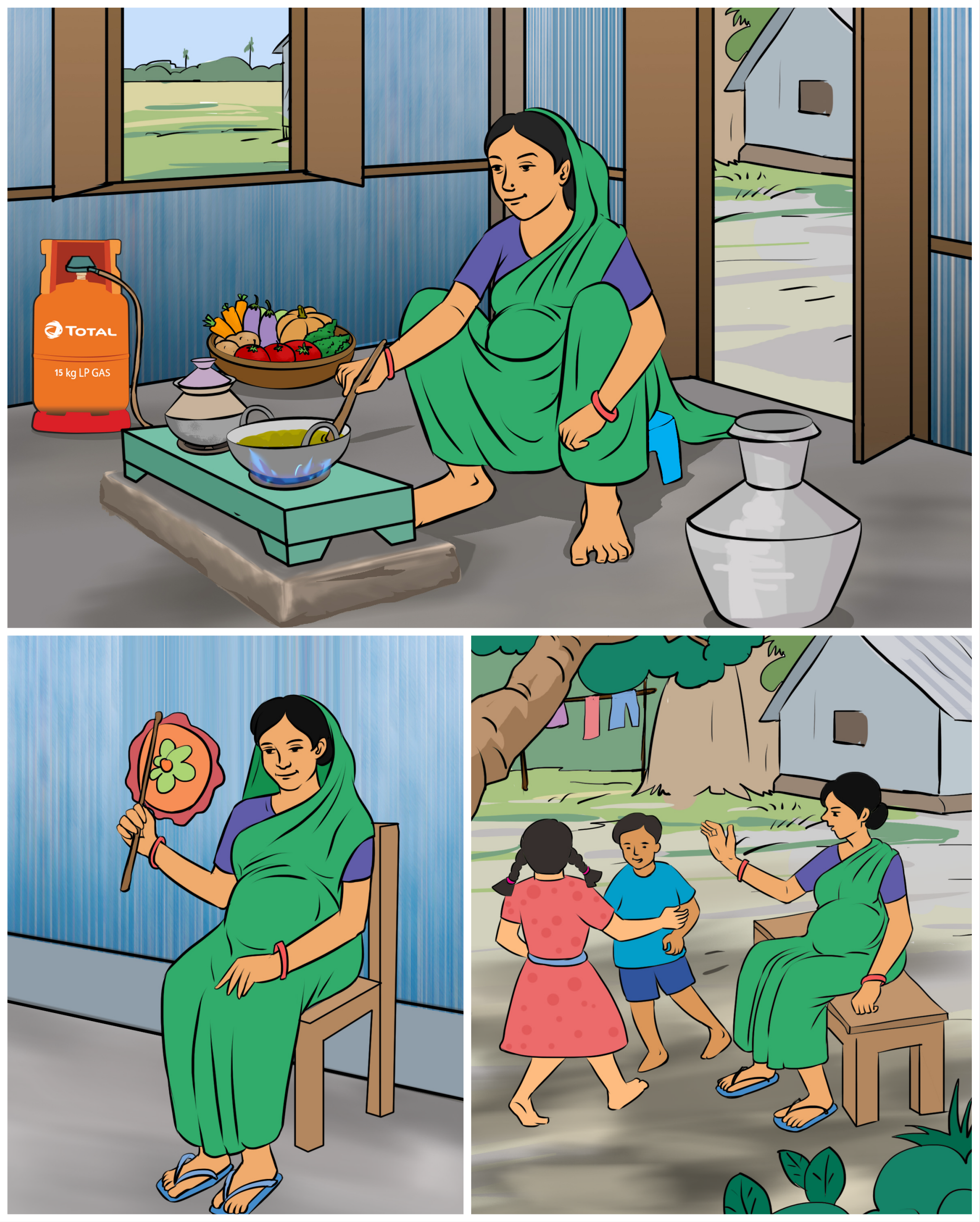

গর্ভাবস্থায় গ্যাসের চুলায় কম সময়ে রান্না করে আপনি আরো বেশি বিশ্রাম নিতে পারবেন। এ সময়ে ছোট বাচ্চা ও বয়স্কদের ভালোভাবে দেখাশোনা করতে পারবেন।

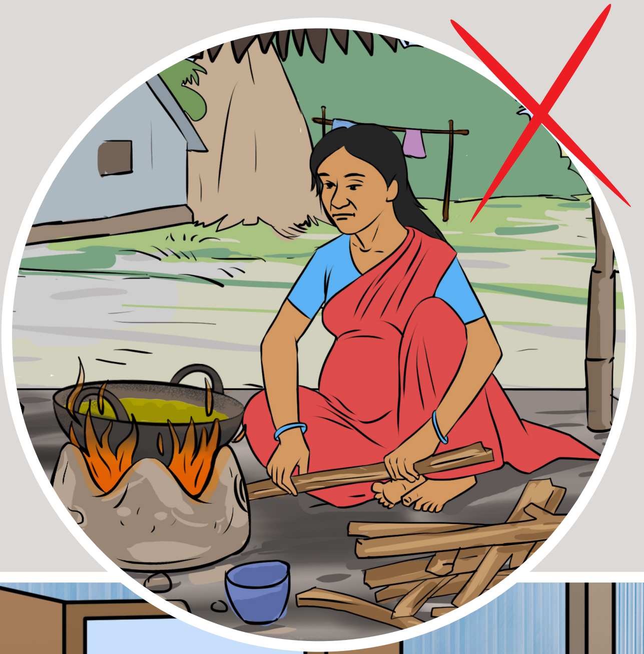

মনে রাখবেন,
গর্ভকালীন সময়ে
কোন কাজেই
গ্যাসের চুলা ব্যতীত
অন্য কোন চুলা
ব্যবহার করবেন
না

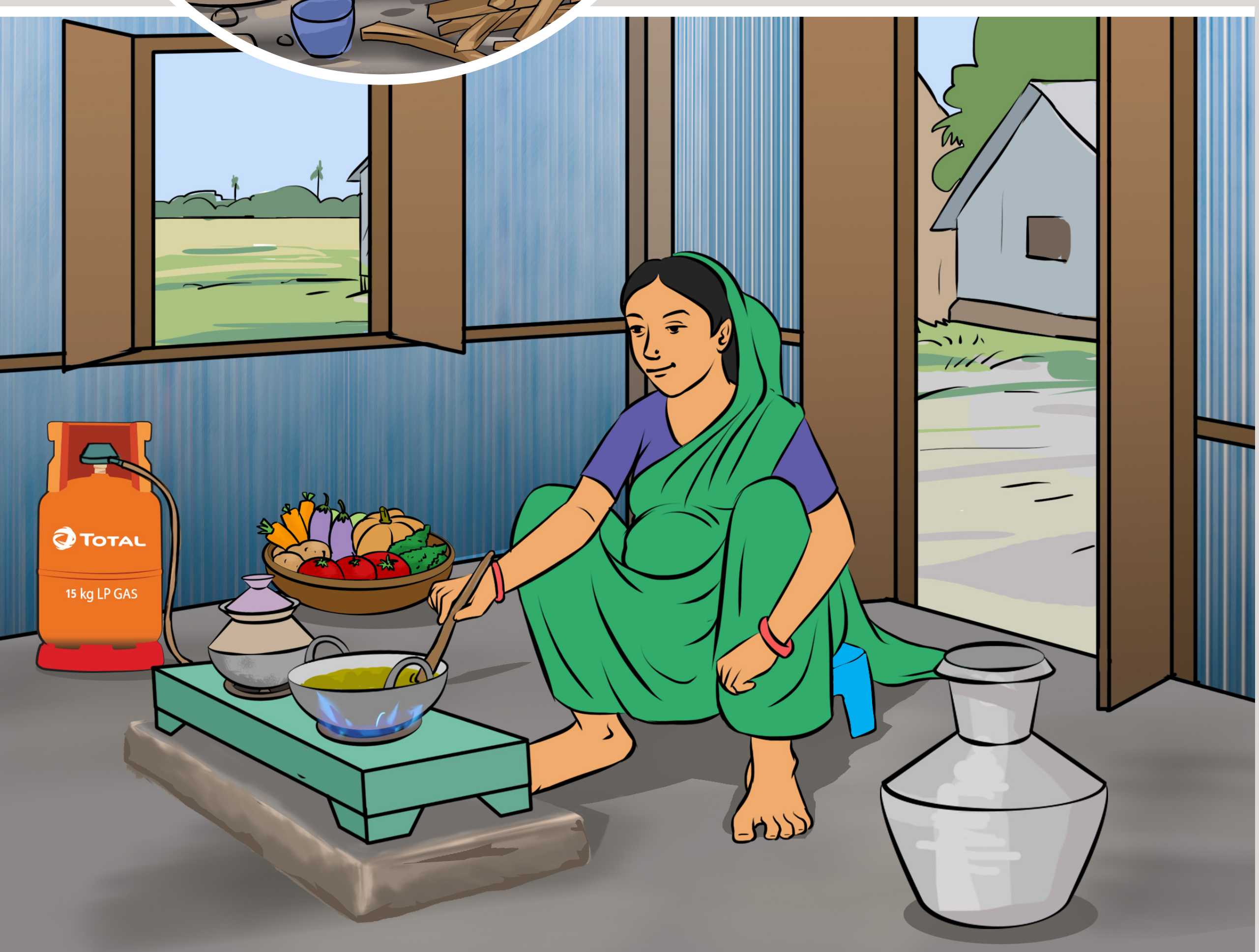

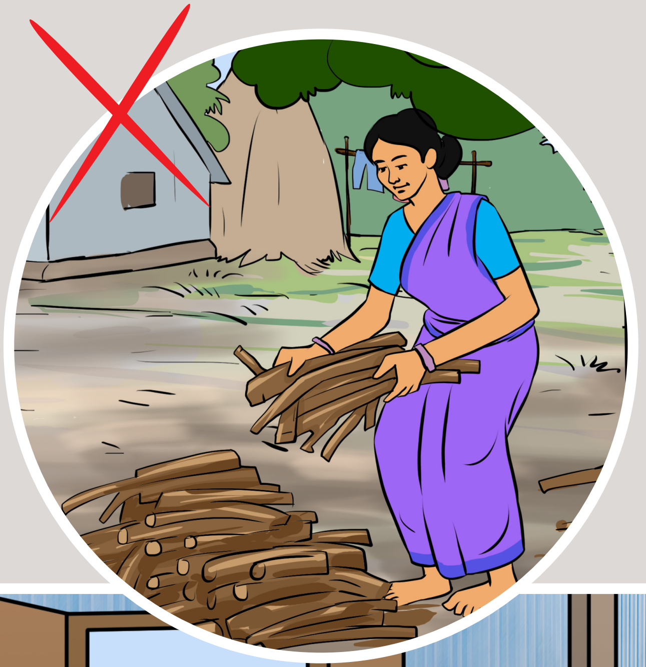

শুধুমাত্র গ্যাসের চুলা ব্যবহার করলে প্রতিদিনের জ্বালানী জোগাড় করা এবং জমা রাখার ঝামেলা থেকে মুক্ত থাকা যায়

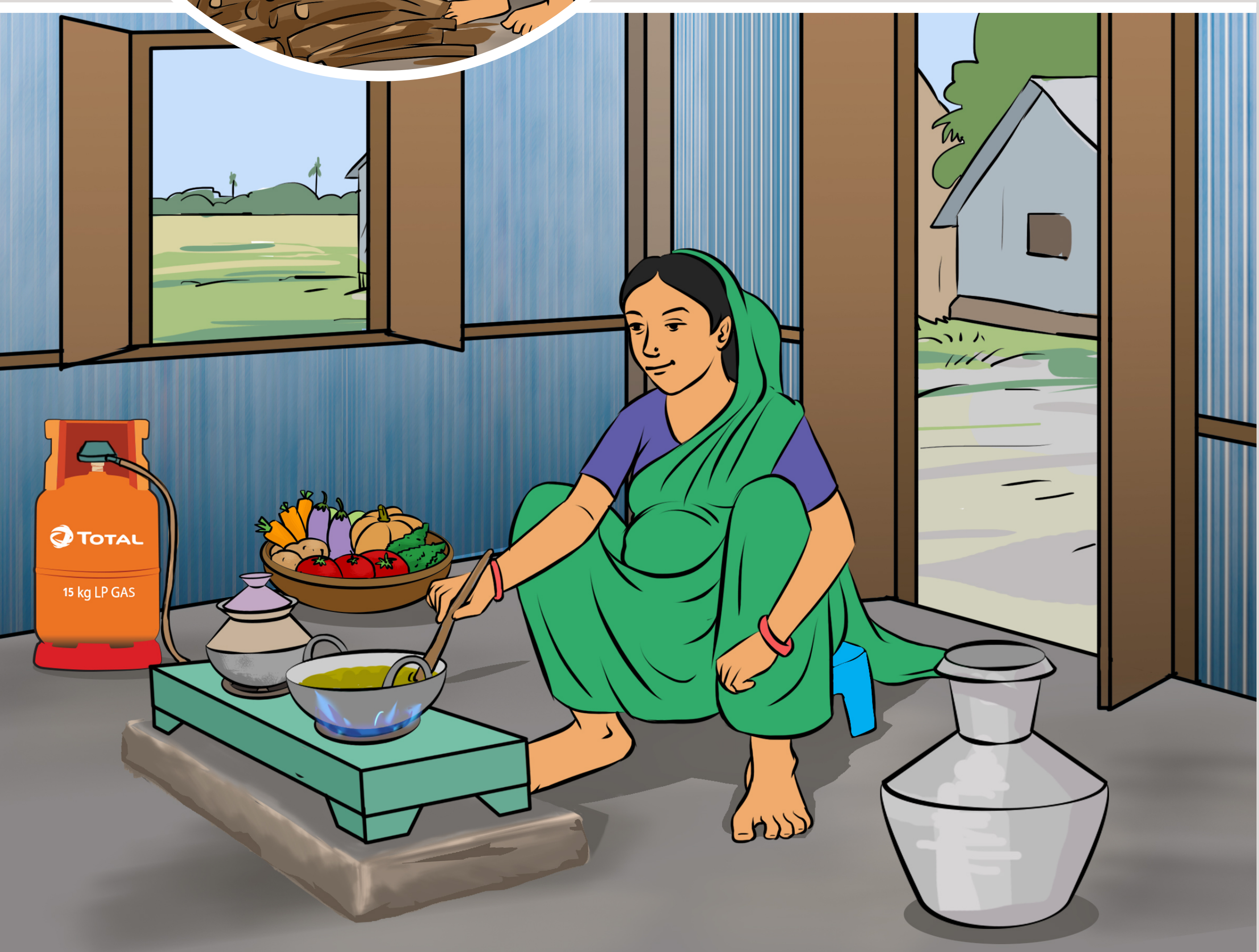

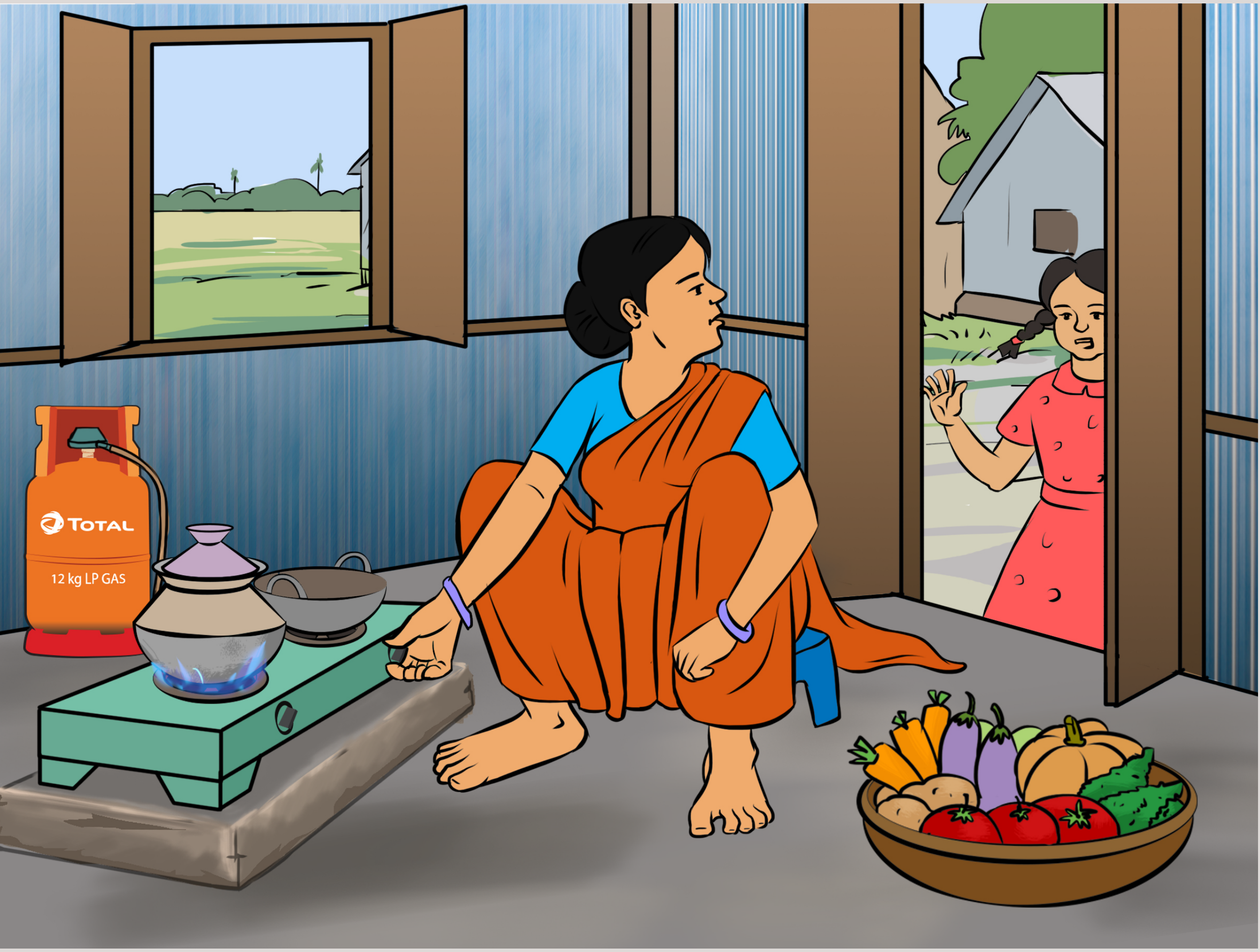

গ্যাসের চুলা জ্বালানো এবং তাপ কমানো বাড়ানো সহজ, তাই আপনি যে কোন সময় চুলা বন্ধ করে রান্না থেকে বিরতি নিতে পারেন

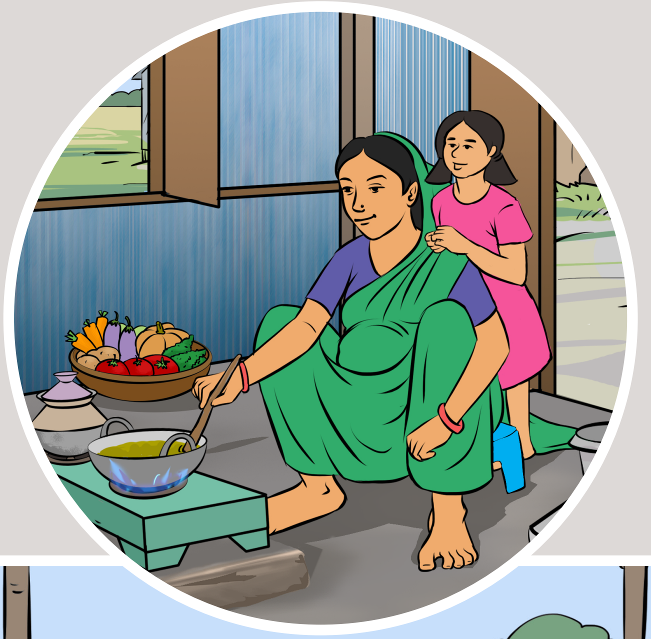

আপনার গ্যাসের চুলা থেকে ধোঁয়া বের হয় না; তাই শ্বাসকষ্ট, চোখ জ্বালা-পোড়া কিংবা কাশি হয় না

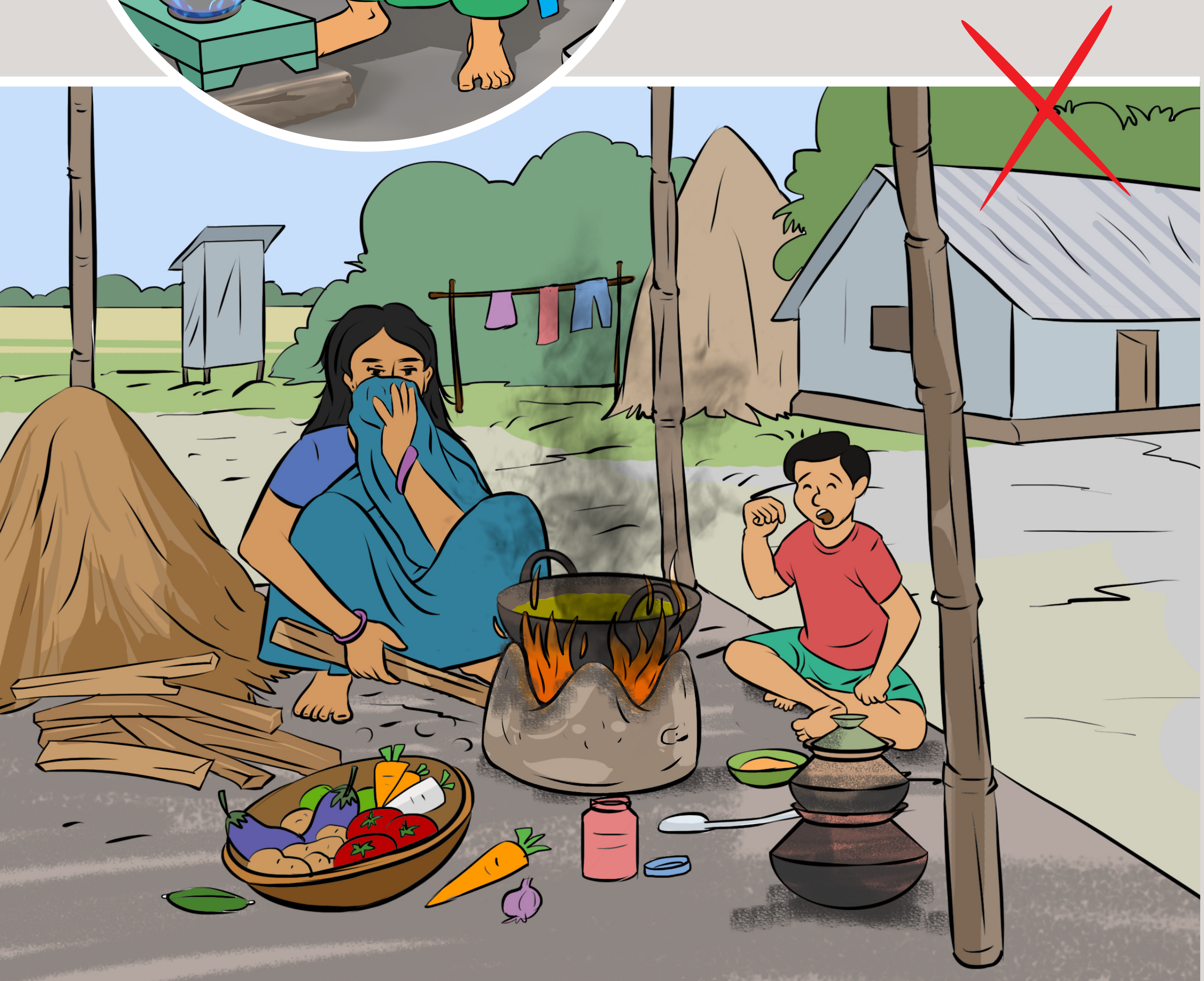

Supplement: online supplemental file 2 [file bmjgh-11-2-s005.pdf]
