# Supplementary material for: Impact of cooking with liquefied petroleum gas compared with traditional cooking practices on perinatal and early neonatal mortality: the Poriborton cluster randomised controlled trial
Source: BMJ Glob Health. 2026 Feb 16;11(2):e020391. doi: 10.1136/bmjgh-2025-020391 (PMC12911768; doi:10.1136/bmjgh-2025-020391)
Supplement: online supplemental file 3 [file bmjgh-11-2-s006.docx]

### BMJ Global Health Author Reflexivity Statement

Adapted from Morton, B., Vercueil, A., Masekela, R., Heinz, E., Reimer, L., Saleh, S., Kalinga, C., Seekles, M., Biccard, B., Chakaya, J., Abimbola, S., Obasi, A. and Oriyo, N. (2022), Consensus statement on measures to promote equitable authorship in the publication of research from international partnerships. Anaesthesia, 77: 264-276. <https://doi.org/10.1111/anae.15597>

| **Study conceptualisation** | |
| --- | --- |
| 1. How does this study address local research and policy priorities? | The Poriborton trial was designed in close collaboration with local stakeholders in Bangladesh to address the pressing issue of perinatal mortality, a key public health concern identified by national health authorities. The study aligns with Bangladesh’s strategic priorities to reduce maternal and neonatal mortality and improve household air quality, particularly in low-resource settings where biomass fuel use is prevalent. |
| 1. How were local researchers involved in study design? | Local researchers were integral to the conceptualisation and design of the Poriborton trial. From the outset, Bangladeshi collaborators contributed to identifying the research questions, selecting appropriate methodologies, and ensuring cultural and contextual relevance. Their insights shaped the intervention design and implementation strategies to ensure feasibility and acceptability within the local context. |
| **Research management** | |
| 1. How has funding been used to support the local research team(s)? | Funding from the NHMRC supportted the salaries of local research staff, capacity-building activities, and infrastructure development. This included training programs, equipment procurement, and administrative support to ensure the sustainability of research capacity within the local institutions. |
| **Data acquisition and analysis** | |
| 1. How are research staff who conducted data collection acknowledged? | Field staff and data collectors have been acknowledged in all publications and presentations arising from the Poriborton trial. Where appropriate, key contributors have been included as co-authors, in line with ICMJE authorship criteria. |
| 1. How have members of the research partnership been provided with access to study data? | All research collaborators, including those based in Bangladesh, have had full access to the study data. Data sharing agreements were established to ensure equitable access and use, and data management systems were designed to facilitate secure and timely sharing. |
| 1. How were data used to develop analytical skills within the partnership? | Capacity-building and ongoing mentorship were provided to local researchers to enhance data analysis skills. Collaborative sessions are ongoing. Junior researchers have been and continue to be supported to lead specific analyses and contribute to manuscript development. |
| **Data interpretation** | |
| 1. How have research partners collaborated in interpreting study data? | Interpretation of findings has been a collaborative process involving regular meetings, joint data review sessions, and iterative feedback loops. Local partners provided critical contextual insights that informed the interpretation of results and their implications for policy and practice. |
| **Drafting and revising for intellectual content** | |
| 1. How were research partners supported to develop writing skills? | Writing sessions and mentorship were provided to early-career researchers, with a focus on manuscript preparation, responding to peer review, and navigating the publication process. Co-authorship was used as a platform for skill development and recognition. |
| 1. How will research products be shared to address local needs? | Findings will be disseminated through local stakeholder meetings, policy briefs, and community engagement events. Materials will be translated into local languages and tailored to different audiences, including policymakers, healthcare providers, and community members. |
| **Authorship** | |
| 1. How is the leadership, contribution and ownership of this work by LMIC researchers recognised within the authorship? | LMIC researchers have held lead and senior authorship positions across multiple publications from the Poriborton project, since we started collaborating in 2013. Authorship decisions were made transparently, based on contributions, and in alignment with international authorship guidelines. |
| 1. How have early career researchers across the partnership been included within the authorship team? | Early career researchers from both LMIC and HIC institutions were actively mentored and included as co-authors. They led specific components of the research and manuscript writing, fostering their professional development and visibility. |
| 1. How has gender balance been addressed within the authorship? | The project supported formal and informal training opportunities, including workshops on research methods, data analysis, and scientific writing. Several LMIC team members pursued higher degrees and advanced their research careers through their involvement in the project.  equity was a key consideration in team composition and authorship. Women researchers were encouraged and supported to take on leadership roles and were well-represented among the authorship team across publications. |
| **Training** | |
| 1. How has the project contributed to training of LMIC researchers? | The project supported formal and informal training opportunities, including workshops on research methods, data analysis, and scientific writing. Several LMIC team members pursued higher degrees and advanced their research careers through their involvement in the project. |
| **Infrastructure** | |
| 1. How has the project contributed to improvements in local infrastructure? | The project invested in strengthening local research infrastructure, including data collection systems, , and skills. These improvements have had lasting benefits beyond the scope of the Poriborton trial. |
| **Governance** | |
| 1. What safeguarding procedures were used to protect local study participants and researchers? | Ethical approvals were obtained from relevant local and ethics committees. Comprehensive training on research ethics, informed consent, and participant confidentiality was provided. Safety protocols were implemented to protect both participants and field staff, particularly in remote and resource-limited settings. |
